# Systematic comparison of published host gene expression signatures for bacterial/viral discrimination

**DOI:** 10.1186/s13073-022-01025-x

**Published:** 2022-02-21

**Authors:** Nicholas Bodkin, Melissa Ross, Micah T. McClain, Emily R. Ko, Christopher W. Woods, Geoffrey S. Ginsburg, Ricardo Henao, Ephraim L. Tsalik

**Affiliations:** 1 Duke University Trinity College of Arts and Sciences, Durham, NC USA; 2 Duke University School of Medicine, Durham, NC USA; 3 Duke Center for Applied Genomics and Precision Medicine, Duke University School of Medicine, Durham, NC USA; 4 Division of Infectious Diseases, Department of Medicine, Duke University School of Medicine, Durham, NC USA; 5 Durham VA Health Care System, Durham, NC USA; 6 Durham Regional Hospital, Durham, NC USA; 7 All of Us Research Program, National Institutes of Health, Bethesda, MD USA; 8 Duke University Department of Biostatistics and Informatics, Durham, NC USA

**Keywords:** Biomarkers, Infectious disease, Diagnostics, Gene expression, Machine learning

## Abstract

**Background:**

Measuring host gene expression is a promising diagnostic strategy to discriminate bacterial and viral infections. Multiple signatures of varying size, complexity, and target populations have been described. However, there is little information to indicate how the performance of various published signatures compare to one another.

**Methods:**

This systematic comparison of host gene expression signatures evaluated the performance of 28 signatures, validating them in 4589 subjects from 51 publicly available datasets. Thirteen COVID-specific datasets with 1416 subjects were included in a separate analysis. Individual signature performance was evaluated using the area under the receiving operating characteristic curve (AUC) value. Overall signature performance was evaluated using median AUCs and accuracies.

**Results:**

Signature performance varied widely, with median AUCs ranging from 0.55 to 0.96 for bacterial classification and 0.69–0.97 for viral classification. Signature size varied (1–398 genes), with smaller signatures generally performing more poorly (*P <* 0.04). Viral infection was easier to diagnose than bacterial infection (84% vs. 79% overall accuracy, respectively; *P* < .001). Host gene expression classifiers performed more poorly in some pediatric populations (3 months–1 year and 2–11 years) compared to the adult population for both bacterial infection (73% and 70% vs. 82%, respectively; *P* < .001) and viral infection (80% and 79% vs. 88%, respectively; *P* < .001). We did not observe classification differences based on illness severity as defined by ICU admission for bacterial or viral infections. The median AUC across all signatures for COVID-19 classification was 0.80 compared to 0.83 for viral classification in the same datasets.

**Conclusions:**

In this systematic comparison of 28 host gene expression signatures, we observed differences based on a signature’s size and characteristics of the validation population, including age and infection type. However, populations used for signature discovery did not impact performance, underscoring the redundancy among many of these signatures. Furthermore, differential performance in specific populations may only be observable through this type of large-scale validation.

**Supplementary Information:**

The online version contains supplementary material available at 10.1186/s13073-022-01025-x.

## Background

Infectious diseases caused an estimated eight million deaths worldwide and 420 million disability-adjusted life years lost in 2019 alone [1]. While most respiratory infections are caused by viral pathogens, up to 75% of all ambulatory care visits result in an antibiotic prescription [2, 3]. This discrepancy is primarily due to a lack of fast and accurate diagnostic methodologies to distinguish bacterial from viral etiologies. Given these diagnostic limitations and the clinical significance of undertreating a bacterial infection, there is a substantial burden of inappropriate antimicrobial overuse. The high rates of antibiotic usage drive antimicrobial resistance, which the Centers for Disease Control and Prevention (CDC) deems as one of the greatest global public health challenges of our time [4–6].

Host gene expression biomarkers offer one solution to address this diagnostic uncertainty. Multiple research groups have described gene expression signatures that discriminate bacterial from viral infection [7–30]. Published signatures vary in size, methods for discovery and validation, and the target clinical populations (e.g., illness severity, site of infection, age, etiology). However, to date, there has been no systematic comparison of these signatures to each other. It is also unclear how these signatures perform once stratified by various population-level differences.

In this study, we identified 28 published gene signatures and validated them in 51 publicly available datasets comprised of 4589 patients [7–30]. This study had two primary aims. The first was to understand how the signatures compare to each other with respect to composition and performance. The second was to define the impact of clinical and demographic characteristics on gene expression-based classification. In addition, thirteen COVID-19-related datasets comprised of 1416 subjects were included to specifically assess signature performance for this infection.

## Methods

### Identification of gene signatures

Herein, we use the term “signature” to describe a set of differentially expressed genes that discriminate phenotypic groups. The term “model” is used to describe a mathematical equation incorporating gene expression data to assign subjects to a given phenotypic group. Since we used a uniform strategy to generate models for all signatures in this analysis, the terms “signature” and “model” may be used interchangeably.

A comprehensive search was performed to identify published host gene expression signatures that differentiate bacterial and viral infection. The search was carried out in PubMed using terms including (Bact* or Vir*) AND (gene expression OR host gene expression OR signature). The last search was performed on October 23, 2021. The citations of any relevant manuscripts were used to identify additional signatures that were missed in the search. The search resulted in 24 publications [7–30], each with a unique list of genes comprising their signature. Four publications identified two gene lists, which were both evaluated.

### Identification of validation datasets

Transcriptome studies, consisting of microarray or complete RNA sequencing data, were systematically reviewed and selected from the Gene Expression Omnibus (GEO) and ArrayExpress with an approach similar to that outlined in the Preferred Reporting Items for Systematic Reviews and Meta-Analyses (PRISMA) statement (Additional file 1: Fig. S1). For standardization in the processing of RNA sequencing data, only studies with raw sequencing data available were included. To avoid incorporation bias, datasets used to develop a given gene expression signature were excluded from its validation in this study. Four of the identified studies used more than one microarray or RNA sequencing platform, so they were partitioned into multiple, single-platform datasets. This resulted in forty-nine microarray datasets and two RNA sequencing datasets.

Once the pool of relevant studies was identified, we manually reviewed each subject from each study. Subjects were excluded from analysis for the following reasons: gene expression data was not generated using either whole-blood or PBMCs, a clinical adjudication did not accompany data, the infectious process was not specified as bacterial or viral, co-infection, serial samples beyond the first time point, and samples from immunocompromised individuals.

With limited publicly available COVID-19 gene expression datasets at the time these experiments were performed, the inclusion criteria were relaxed for COVID-specific analyses. Studies that sequenced nasopharyngeal swabs and tissue biopsies were included, as well as one study with fewer than ten subjects. RNA sequencing studies that did not provide raw sequencing data were also included.

### Case definitions

Each subject was annotated with clinical phenotype, pathogen, age, race, ethnicity, and ICU status based on the metadata accompanying the entry in GEO or ArrayExpress or as described in the accompanying published citation. Subjects were classified as one of four clinical phenotypes: bacterial infection, viral infection, healthy, or non-infectious illness [including Systemic Inflammatory Response Syndrome (SIRS)]. Subjects annotated as ICU patients were admitted to an ICU or critical care unit, or they were identified as receiving ECMO or mechanical ventilation. The group of subjects annotated with “non-ICU” do not include subjects identified as healthy. Racial annotations were classified into four groups: Asian, Black, White, and others. Age was classified into five distinct groups: ≤3 months (neonate), 3 months to 2 years (infant), 2 years to 12 years (child), 12 years to 18 years (adolescent), and >18 years (adult). Since there were only 60 cases of bacterial/viral co-infections, they were excluded from analysis. Standardized annotations for each subject in this study can be found in Additional file 2: Table S6.

### Gene expression data processing

Pre-processed microarray data from the selected studies and samples were downloaded and parsed with various open-source Python packages. Probes from each validation dataset were converted into Ensembl IDs using g:Profiler version e99_eg46_p14_f929183 [31] and matched with the gene IDs in each signature to generate signature-specific gene expression for each patient (Additional file 3: Table S7). Duplicate genes and the genes that could not be matched to an Ensemble ID were removed from the signatures validated in this study. Multiple probes for a given gene were left as distinct features in the model.

Raw RNA sequencing data from GEO datasets were processed and downloaded using GREIN [32]. For datasets not published in GEO or those that did not include raw data, count files were located and downloaded directly for normalization and analysis. All RNA sequencing datasets were normalized using trimmed mean of *M* value (TMM), followed by counts per million (CPM) in the edgeR package [33, 34].

### Statistical analysis

Each gene signature was validated independently in all datasets as a binary classifier for bacterial vs. non-bacterial infection (viral, healthy, or non-infectious illness) and viral vs. non-viral infection (bacterial, healthy, or non-infectious illness). The entire gene panel of a signature was evaluated in each binary classifier. Models were fit for each signature in each dataset using logistic regression with a lasso penalty, and performance was evaluated using nested leave-one-out cross-validation in scikit-learn [35]. In some cases, performance of the composite signature (“All”) in datasets with more than 300 subjects was evaluated using nested five-fold cross-validation to minimize compute time (GSE152075, GSE73461, and GSE61821). Creating dataset-specific models overcomes batch effects since each signature is optimized in each dataset. Coefficients of each logistic regression were recorded for analysis of relative gene importance. The code utilized for cross-validation is in Additional file 4.

Signature performance was characterized by the weighted mean of a signature’s area under the receiving operating characteristic curve (AUC) across all validation studies. Values were weighted based on the number of subjects in a validation dataset. Median AUC and IQR were used to summarize the distribution of weighted mean AUCs for subsets of validation studies. Dataset-specific thresholds were used to measure signature accuracy and generate confusion matrices. Thresholds were determined by the maximization of Youden’s J-statistic [36]. 95% confidence intervals of weighted means, accuracies, positive predictive values (PPV), and negative predictive values (NPV) were generated by bootstrapping with 1000 iterations. Signature hierarchical summary receiving operating characteristic (HSROC) curves were generated using the Rutter and Gatsonis HSROC model in Stata’s metandi package [37, 38]. Sensitivity, specificity, and diagnostic odds ratio (DOR) values and confidence intervals were generated using the bivariate model in Stata’s metandi package [38, 39]. Heterogeneity in DOR values were measured using the Mantel-Haenszel method in the meta R package [40, 41].

Relative gene importance was determined by analysis of logistic regression coefficients from each fold of the composite signature’s leave-one-out cross-validation. Importance was characterized by the average of each gene’s coefficient in all models. For genes that mapped to multiple microarray probes, the coefficient with the largest magnitude was used for the average.

Determinations of significance included Wilcoxon rank-sum and Kruskal-Wallis tests. Corrections for multiple testing and significance cutoffs were performed with the Benjamini/Hochberg method (*α* = 0.05) [42]. Determinations of significance between multiple patient groups were made using a reference group specific to the category: “Adult” for age comparisons, “All Bacterial” or “All Viral” for pathogen comparisons, “All Subjects” with race data for race comparisons, “Not Hispanic or Latino” for ethnicity comparisons, and “non-ICU” for infection severity comparisons. Correlation coefficients and their associated determinations of significance were computed with Pearson’s correlation.

## Results

### Gene expression signatures

**Fig. 1:**
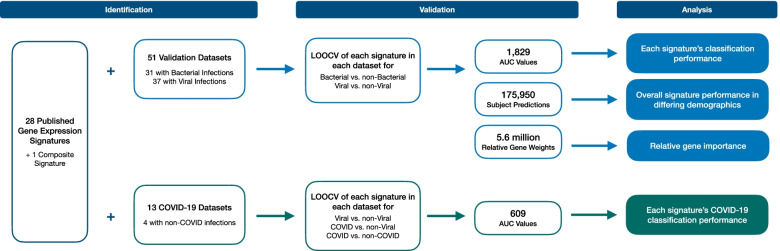
Study flow diagram. The performance of 28 published gene expression signatures and one composite signature was evaluated using leave-one-out cross-validation (LOOCV) in 51 publicly available datasets. LOOCV was performed for both bacterial vs. non-bacterial classification and viral vs. non-viral classification. LOOCV was also performed to measure the performance of signatures in 13 publicly available COVID-19 datasets. Performance was then measured by area under the receiving operating characteristic curve (AUC) values and individual subject predictions. Relative gene importance was characterized by the relative gene weights in each generated model

We identified 28 [7–30] published gene expression signatures that were shown to discriminate bacterial and viral infection. The study flow diagram in Fig. 1 depicts our methodology for validating these signatures. The signatures differed in size (1–398 genes by Ensembl ID) and the demographics of subjects used for signature discovery (Table 1, Additional file 5: Table S8). In twenty-three cases, signatures were discovered in cohorts that included both bacterial and viral phenotypes. In the remaining five cases, the discovery cohorts did not include bacterial infections, but the validation cohorts did, so these signatures were included. Five published signatures were each a subset of other, larger signatures, in which case both were included. In total, there were 864 unique genes identified across all signatures.

**Table 1:** Characterization of the 28 identified host gene expression signatures

Signature code	Publication first author	Publication last author	Number of genes	Discovery age group	Discovery phenotypes
TS1 [7]	Tang	Schughart	1	Adults	Viral (influenza), healthy
HL2^a^ [8]	Herberg	Levin	2	Pediatrics	Bacterial, viral
LC2 [9]	Lei	Chen	2	All	Bacterial, viral, healthy, SIRS
XW2 [10]	Xu	Wang	2	All	Bacterial, viral, healthy, SIRS
GS3 [11]	Gomez-Carballo	Salas	3	All	Bacterial, viral
LS3 [12]	Li	Sriskandan	3	Adults	Viral (w/ COVID), bacterial, SIRS
SB4^a^ [13]	Sampson	Brandon	4	All	Viral, SIRS
SK7^a^ [14]	Sweeney	Khatri	7	All	Bacterial, viral, SIRS
SB8 [13]	Sampson	Brandon	8	All	Viral, SIRS
RC10 [15]	Ravichandran	Chandra	10	All	Bacterial, viral, healthy
SN10 [16]	Sampson	Noursadeghi	10	All	Bacterial, viral, healthy, SIRS
SR10 [17]	Suarez	Ramilo	10	Adults	Bacterial, viral, co-infection, healthy
AK11 [18]	Andres-Terre	Khatri	11	All	Bacterial, viral, healthy, SIRS
BF11 [19]	Bhattacharya	Falsey	11	Adults	Bacterial, viral
NC19 [20]	Ng	Chiu	19	Adults	Viral (w/ COVID), bacterial, healthy
SL20 [21]	Song	Lei	20	All	Bacterial, viral, healthy, SIRS
MW23 [22]	McClain	Woods	23	All	Viral (w/ COVID), bacterial, healthy
ZG25^a^ [23]	Zaas	Ginsburg	25	Adults	Viral, healthy
MS29 [24]	Mayhew	Sweeney	29	All	Bacterial, viral, healthy, SIRS
PT29 [25]	Parnell	Tang	29	Adults	Bacterial, viral, healthy, SIRS
RC31 [26]	Ramilo	Chaussabel	31	Pediatrics	Bacterial, viral
HS33 [27]	Hu	Storch	33	Infants	Bacterial, viral, healthy
HL34 [8]	Herberg	Levin	34	Pediatrics	Bacterial, viral
ZG48 [28]	Zaas	Ginsburg	48	Adults	Viral, healthy
MR59 [29]	Mahajan	Ramilo	59	Neonates	Bacterial, viral, healthy
TW96 [30]	Tsalik	Woods	96	Adults	Bacterial, viral, SIRS
MW139 [22]	McClain	Woods	139	All	Viral (w/COVID), Bacterial, healthy
AK398 [18]	Andres-Terre	Khatri	398	All	Bacterial, viral, healthy, SIRS
All	-	-	864	-	-

Published host gene expression signatures varied in size and discovery cohort characteristics. Signatures were named using the first and last author’s initials, followed by the number of unique genes in the signature. Neonates include subjects <3 months of age; infants include subjects <3 years of age; Pediatrics includes subjects <18 years of age. ^a^Signature is a subset of another published signature

While some signatures were generated to broadly differentiate between bacterial and viral infection, many signatures were targeted for specific etiologies or patient populations. Twelve signatures were developed for respiratory illness, five for pediatric patients, eight to identify sepsis, four to identify influenza, and three to identify COVID-19. Most signatures were developed from peripheral whole blood; however, six utilized purified PBMCs in their discovery cohort, one used NP swabs, and another used isolated leukocytes. Finally, nine of the signatures were generated solely by analysis of publicly available data.

### Validation datasets

To build a validation cohort, we performed a systematic search of publicly available gene expression datasets in GEO and ArrayExpress (Fig. 1, Additional file 1: Fig. S1). The search identified 506 potential records; however, only 47 studies met our inclusion criteria. The 47 studies comprised 4589 whole-blood samples that belonged to subjects with clinically adjudicated bacterial or viral infections and healthy or non-infectious illness controls. Subjects varied by age, pathogen class, and infection severity (Additional file 6: Table S9, Additional file 1: Table S1).

Four of the validation studies contained samples from more than one microarray platform. These datasets were separated and treated independently, resulting in 51 total datasets. Thirty-one of these datasets included bacterial infections and could be used to evaluate bacterial classification performance. Similarly, 37 included viral infections and could be used to evaluate viral classification performance. Gene expression was measured by either commercially available microarrays (*n*=49) or by complete RNA sequencing (*n*=2).

### Gene importance

All the signatures were developed or validated for their ability to discriminate bacterial and viral infection. However, other clinical groups were variably included in these studies, such as healthy controls or those with non-infectious illness. We therefore generated two predictive models: bacterial vs. non-bacterial infection and viral vs. non-viral infection to accommodate all four clinical groups.

To identify genes that were most important to bacterial and viral classification, we first looked at the frequency with which each gene appeared in a signature’s gene list. After excluding five signatures that were subsets of larger signatures, we found 71 common genes that were present in ≥2 signatures (Additional file 3: Table S7). *IFI27* was the most common, found in 12 of the 23 unique signatures, followed by *ISG15* in 8 signatures and *RSAD2*, *OASL*, and *IFI44L* in 7 signatures.

We then assessed the relative importance of each gene for bacterial and viral classification. To do so, we created a composite signature comprised of the 864 unique genes identified in at least one published signature. In this composite signature, each gene would have equal representation in the model validation. The composite signature was then used to build predictive models for bacterial vs. non-bacterial classification and viral vs. non-viral classification in each validation dataset. Based on their average logistic regression coefficients, the five most important genes for bacterial classification were *CEPT* (+), *RPGRIP1* (-), *FCER1A* (-), *IFI27* (-), and *PDE9A* (-), where plus indicates upregulation and minus indicates downregulation for bacterial infection. Similarly, for viral classification, the five most important genes were *IFI27* (+), *OTOF* (+), *FCER1A* (-), *LARP1* (+), and *OAS1* (+) (Additional file 1: Table S2).

### Comparison of gene expression signatures

Although various machine-learning methods were used to develop the 28 published signatures, we used a single, standardized strategy to train and validate the models. Specifically, we performed nested, leave-one-out cross-validation on the 28 signatures for each of the 51 validation datasets. Furthermore, there were four clinical phenotypes included in the validation datasets: bacterial infection, viral infection, healthy, and non-infectious illness [including Systemic Inflammatory Response Syndrome (SIRS)]. We therefore developed two binary models: bacterial vs. non-bacterial etiologies and viral vs. non-viral etiologies.

**Fig. 2:**
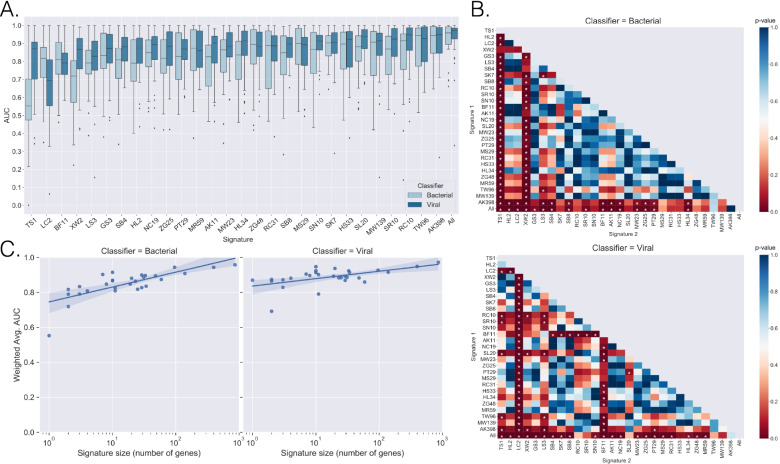
Signature classification performance. **A** Box-plots were generated for each signature’s AUCs as measured across the validation datasets for bacterial vs. non-bacterial and viral vs. non-viral classification. **B** Signature AUC distributions were compared against each other with the Wilcoxon rank-sum test, and *p*-values were plotted in a heatmap for bacterial classification (top) and viral classification (bottom). *P*-values were corrected for multiple comparisons with the Benjamini/Hochberg method. * indicates *p*-value ≤ 0.05. **C** Linear regression was applied to the relationship between the number of genes in a signature (log-transformed) and the signature’s median AUC across the validation datasets for bacterial classification (left) and viral classification (right)

We calculated the AUC for each signature in each of the validation datasets. For bacterial classification, the median AUC for all published signatures was 0.86 (range across all signatures was 0.55–0.96). For viral classification, the median AUC was significantly higher at 0.89 (range across all signatures was 0.69–0.97) (*P* < 0.001, Fig. 2a).

A global comparison of signatures found statistically significant performance differences between the signatures for bacterial classification (*P* < 0.001) and viral classification (*P* < 0.001) (Fig. 2b). We observed that larger signatures correlated with better performance for both bacterial classification (*r* = 0.417; *P* = 0.024) and viral classification (*r* = 0.416; *P* = 0.025) across the 29 signatures evaluated (Fig. 2c). Additional signature-based performance metrics are represented in Table 2, Additional file 1: Table S3, and Additional file 1: Fig. S2. Dataset-based performance metrics are presented in Additional file 1: Fig. S3 and Additional file 6: Table S9.

**Table 2:** Summarized performance of gene signatures in bacterial and viral classification

Signature	Bacterial vs. non-bacterial				Viral vs. non-viral			
	Weighted Mean AUC (95% CI)	Sensitivity (95% CI)	Specificity (95% CI)	DOR (95% CI)	Weighted Mean AUC (95% CI)	Sensitivity (95% CI)	Specificity (95% CI)	DOR (95% CI)
TS1	0.547 [0.445–0.658]	0.825 [0.74–0.887]	0.592 [0.473–0.702]	7 [5–10]	0.826 [0.729–0.892]	0.872 [0.813–0.914]	0.867 [0.806–0.911]	44 [25–79]
HL2	0.778 [0.667–0.857]	0.812 [0.723–0.877]	0.862 [0.787–0.913]	27 [14–52]	0.861 [0.803–0.903]	0.81 [0.74–0.865]	0.905 [0.84–0.945]	41 [21–80]
LC2	0.764 [0.652–0.843]	0.873 [0.786–0.928]	0.799 [0.706–0.868]	27 [14–54]	0.641 [0.55–0.737]	0.808 [0.709–0.88]	0.7 [0.558–0.812]	10 [6–17]
XW2	0.690 [0.608–0.763]	0.741 [0.63–0.828]	0.803 [0.711–0.871]	12 [7–19]	0.851 [0.775–0.894]	0.815 [0.747–0.867]	0.874 [0.824–0.911]	30 [17–53]
GS3	0.796 [0.655–0.910]	0.836 [0.75–0.897]	0.92 [0.852–0.958]	58 [23–147]	0.843 [0.767–0.901]	0.812 [0.735–0.871]	0.876 [0.813–0.92]	31 [15–62]
LS3	0.764 [0.692–0.838]	0.778 [0.718–0.829]	0.792 [0.705–0.858]	13 [8–21]	0.831 [0.782–0.876]	0.804 [0.752–0.848]	0.849 [0.774–0.903]	23 [13–41]
SB4	0.776 [0.662-0.876]	0.83 [0.743-0.892]	0.792 [0.707–0.858]	19 [10–36]	0.868 [0.817–0.914]	0.807 [0.76–0.846]	0.896 [0.851–0.929]	36 [22–60]
SK7	0.863 [0.812–0.922]	0.861 [0.813–0.898]	0.88 [0.821–0.921]	45 [24–87]	0.901 [0.849–0.936]	0.866 [0.822–0.9]	0.883 [0.829–0.922]	49 [26–91]
SB8	0.835 [0.734–0.914]	0.86 [0.759–0.923]	0.859 [0.778–0.913]	37 [17–83]	0.884 [0.836–0.924]	0.832 [0.788–0.869]	0.884 [0.842–0.915]	38 [23–63]
RC10	0.849 [0.708–0.931]	0.886 [0.817–0.931]	0.909 [0.817–0.958]	78 [27–227]	0.922 [0.871–0.955]	0.892 [0.85–0.923]	0.901 [0.841–0.939]	75 [35–161]
SN10	0.854 [0.758–0.918]	0.868 [0.825–0.901]	0.867 [0.786–0.921]	43 [20–90]	0.897 [0.846–0.937]	0.837 [0.787–0.878]	0.915 [0.872–0.944]	55 [30–103]
SR10	0.844 [0.759–0.909]	0.843 [0.779–0.891]	0.89 [0.839–0.927]	43 [23–82]	0.915 [0.865–0.950]	0.902 [0.86–0.932]	0.903 [0.859–0.934]	85 [42–171]
AK11	0.794 [0.708–0.879]	0.794 [0.703–0.862]	0.875 [0.81–0.92]	27 [14–51]	0.887 [0.826–0.923]	0.849 [0.784–0.897]	0.862 [0.813–0.9]	35 [19–66]
BF11	0.812 [0.742–0.866]	0.85 [0.794–0.892]	0.815 [0.746–0.869]	25 [13–48]	0.801 [0.748–0.843]	0.816 [0.768–0.856]	0.768 [0.706–0.82]	15 [10–22]
NC19	0.832 [0.753–0.897]	0.864 [0.792–0.914]	0.839 [0.784–0.882]	33 [18–61]	0.885 [0.825–0.921]	0.86 [0.819–0.892]	0.858 [0.805-0.899]	37 [21-66]
SL20	0.850 [0.748–0.907]	0.84 [0.78–0.886]	0.88 [0.83–0.917]	38 [18–81]	0.915 [0.868–0.948]	0.899 [0.858–0.929]	0.889 [0.842–0.924]	71 [37–136]
MW23	0.826 [0.732–0.889]	0.874 [0.82–0.914]	0.829 [0.738–0.893]	34 [17–66]	0.894 [0.838–0.933]	0.875 [0.826–0.911]	0.885 [0.839–0.919]	54 [28–105]
ZG25	0.817 [0.716–0.889]	0.843 [0.767–0.898]	0.849 [0.785–0.896]	30 [15–60]	0.882 [0.815–0.926]	0.86 [0.792–0.909]	0.892 [0.849–0.924]	51 [27–95]
MS29	0.873 [0.766–0.938]	0.912 [0.855–0.948]	0.883 [0.796–0.936]	78 [30–206]	0.894 [0.826–0.937]	0.826 [0.754–0.881]	0.885 [0.827–0.925]	37 [19–71]
PT29	0.810 [0.716–0.889]	0.846 [0.769–0.901]	0.837 [0.777–0.884]	28 [15–55]	0.873 [0.821–0.911]	0.827 [0.773–0.87]	0.845 [0.797–0.883]	26 [15–44]
RC31	0.842 [0.755–0.903]	0.868 [0.804–0.913]	0.849 [0.764–0.907]	37 [17–79]	0.891 [0.836–0.927]	0.857 [0.816–0.89]	0.871 [0.815–0.912]	40 [24–67]
HS33	0.854 [0.771–0.913]	0.878 [0.812–0.923]	0.864 [0.796–0.912]	46 [21–98]	0.891 [0.820–0.934]	0.861 [0.796–0.908]	0.893 [0.838–0.931]	52 [26–103]
HL34	0.814 [0.690–0.895]	0.833 [0.752–0.892]	0.871 [0.809–0.915]	34 [17-67]	0.898 [0.831–0.942]	0.871 [0.811–0.915]	0.906 [0.858–0.939]	65 [33–128]
ZG48	0.847 [0.760–0.914]	0.912 [0.841–0.953]	0.883 [0.8–0.935]	78 [32–194]	0.876 [0.799–0.928]	0.846 [0.79–0.889]	0.886 [0.84–0.919]	43 [23–77]
MR59	0.829 [0.724–0.904]	0.909 [0.843–0.949]	0.846 [0.76–0.905]	55 [24–127]	0.864 [0.797–0.918]	0.816 [0.746–0.87]	0.881 [0.833–0.916]	33 [18–61]
TW96	0.844 [0.757–0.921]	0.908 [0.83–0.952]	0.91 [0.843–0.95]	99 [36–271]	0.871 [0.808–0.935]	0.923 [0.867–0.957]	0.898 [0.836–0.938]	106 [39–284]
MW139	0.834 [0.750–0.908]	0.906 [0.835–0.949]	0.869 [0.788–0.922]	64 [28–146]	0.871 [0.808–0.923]	0.887 [0.841–0.921]	0.884 [0.808–0.932]	60 [26–138]
AK398	0.886 [0.820–0.951]	0.939 [0.887–0.968]	0.923 [0.868–0.956]	184 [61–553]	0.896 [0.850–0.946]	0.912 [0.867–0.942]	0.932 [0.866–0.966]	141 [51–392]
All	0.905 [0.842–0.957]	0.927 [0.881–0.956]	0.927 [0.875–0.959]	162 [65–401]	0.933 [0.898–0.965]	0.934 [0.9–0.957]	0.92 [0.872–0.951]	164 [73–369]

Weighted mean AUC, sensitivity, specificity, and diagnostic odds ratio (DOR) for each host gene expression signature are presented. Values were weighted based on the number of subjects in the validation dataset. Sensitivity, specificity, and DOR values and their confidence intervals were calculated using hierarchical summary ROC modeling.

### Overall signature performance in validation datasets

We next evaluated classification performance by stratifying the validation datasets based on pediatric vs. adult enrollment, the number of conditions being classified, and biological source (peripheral blood mononuclear cells vs. whole blood). This evaluation was performed for all published signatures and the composite signature.

**Fig. 3:**
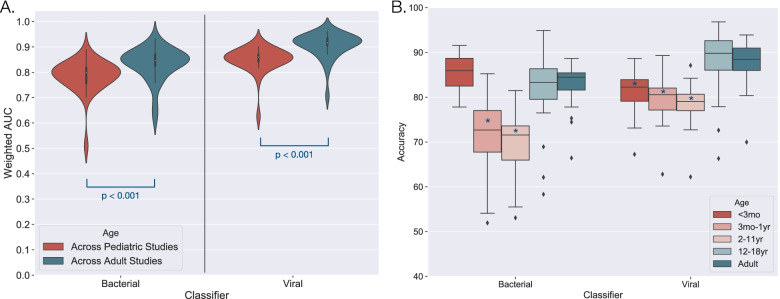
Signature classification performance by age. **A** Weighted mean AUCs were generated for each signature’s classification of bacterial patients and viral patients across pediatric-only (red) and adult-only (blue) datasets. Values were weighted based on the number of subjects in a validation dataset. The distributions of such weighted mean AUCs were plotted, and significance was determined by the Wilcoxon rank-sum test. **B** After pooling samples across datasets, each signature’s accuracy was calculated and plotted for five age groups (<3 months, 3 months–1 year, 2–11 years, 12–18 years, and adult). This plot shows the median and IQR of each signature’s accuracy in each age group for bacterial and viral classification. * indicates *p* < 0.05 as compared to the adult population

Of the 51 validation datasets, 27 were restricted to pediatric subjects (<18 years) and 21 were restricted to adult subjects. Across the evaluated signatures, we observed significantly lower AUCs in pediatric-only studies as compared to adult-only studies (Table 3) (Fig. 3a). This difference was present in both bacterial (0.80 vs. 0.85 median AUCs; *P* < 0.001) and viral classification (0.86 vs. 0.92 median AUCs; *P* < 0.001), and it was not due to differences in platform, dataset size, or the phenotypes represented. We also investigated the possibility that signature discovery population impacted performance. For example, signatures discovered in a pediatric cohort might perform better in pediatric validation datasets as compared to signatures discovered in an adult cohort. However, this did not confer any improvement in classification (bacterial: *P* = 0.747, Viral: *P* = 0.874).

**Table 3:** Overall signature classification performance stratified by dataset characteristics

Parameter	Bacterial vs. non-bacterial				Viral vs. non-viral			
	Median AUC	IQR	*p*-value	N	Median AUC	IQR	*p*-value	N
**All datasets**	0.832	[0.796–0.849]	-	31	0.884	[0.864–0.896]	-	37
**Age**	-	-	-	-	-	-	-	-
Adult only	0.846	[0.826–0.870]	-	14	0.916	[0.906–0.935]	-	12
Pediatric only	0.798	[0.756–0.818]	**< 0.001**	15	0.860	[0.841–0.870]	**< 0.001**	24
**# of phenotypes**	-	-	-	-	-	-	-	-
2 phenotypes	0.871	[0.846–0.894]	-	12	0.911	[0.880–0.923]	-	22
>2 phenotypes	0.819	[0.788–0.835]	**< 0.001**	19	0.855	[0.838–0.874]	**< 0.001**	15
**Biological source**	-	-	-	-	-	-	-	-
Whole blood	0.838	[0.802–0.859]	-	28	0.884	[0.865–0.900]	-	32
PBMC	0.705	[0.668–0.753]	**< 0.001**	3	0.831	[0.791–0.882]	**0.012**	4

AUCs were calculated for each of the 29 evaluated signatures and then stratified by different dataset characteristics. Mean AUCs were first generated for each signature across the datasets in the parameter group, weighted by the number of subjects in each validation dataset. The median of the weighted AUC values and IQR were then calculated and presented here. *N* represents the number of datasets for the specified cohort composition.

Thirty-two validation datasets included only two phenotypic groups, which were some combination of bacterial, viral, healthy, or non-infectious illness (e.g., bacterial and non-infectious illness or bacterial and viral). However, nineteen datasets included three or more of these phenotypes (e.g., bacterial, viral, and non-infectious illness). AUCs were higher when classifying only two clinical phenotypes compared to classification of more than two groups for both bacterial (0.87 vs. 0.82 median AUCs; *P* < 0.001) and viral classification (0.91 vs. 0.85 median AUCs; *P* < 0.001) (Table 3).

Peripheral whole blood was the source of gene expression data in 45 validation datasets. In contrast, five studies used peripheral blood mononuclear cells (PBMCs). We found that AUCs of the signatures were higher in datasets derived from whole blood compared to PBMCs in both bacterial (0.84 vs. 0.70 median AUCs; *P* < 0.001) and viral classification (0.88 vs. 0.83 median AUCs; *P* = 0.01) (Table 3).

### Overall signature performance in distinct patient populations

After evaluating the impact of dataset characteristics on classification, we next evaluated the impact of individual subject characteristics. To do this, we used annotations for each subject as provided by the dataset contributors. These annotations were limited and not available for all subjects in all datasets. However, the most commonly available information focused on subject age, microbiological etiology, race, ethnicity, or illness severity. We established dataset-specific thresholds for each signature and model. After applying these thresholds to each subject, we measured the percentage of signatures that correctly classified the subject. This accuracy metric was pooled across subjects based on age, severity, race, ethnicity, or microbiology.

After stratifying by age, we found that pediatric subjects between 12 and 18 years of age exhibited similar performance to adults for both bacterial and viral classification (Table 4 and Fig. 3b). In contrast, subjects aged 3 months to 11 years exhibited significantly lower accuracy for bacterial classification and viral classification (*P* < 0.001 for both comparisons) (Table 4). While subjects younger than 3 months of age also exhibited lower accuracy for viral classification, this was not true for bacterial classification (Table 4 and Fig. 3b). We examined signature accuracy by pathogen, including those with sufficient representation across studies, and found significant performance differences for some bacterial and viral pathogens (Table 4). Two bacterial pathogens were associated with significantly higher accuracies: *Staphylococcus* (other than *S. aureus*) infections (91%, 95% CI 88–94%) and *Burkholderia pseudomallei* infections (90%, 95% CI 85–93%). Intracellular and extracellular bacterial infections were distinguished equally well from non-bacterial infection (83% and 84% overall accuracy, respectively). For viruses, influenza infections were associated with higher accuracies (89%, 95% CI 87–90%), whereas adenovirus (74%, 95% CI 62–84%) and rhinovirus infections (74%, 95% CI 70–78%) were associated with lower accuracies.

**Table 4:** Overall accuracy of gene signatures in distinct patient populations

Parameter	Bacterial vs. non-bacterial			Viral vs. non-viral		
	Accuracy (%)	*p*-value	*N* (subjects/studies)	Accuracy (%)	*p*-value	*N* (subjects/studies)
**All subjects**	79 (78–80)	-	2887/31	84 (83–85)	-	3584/37
**Pathogen**	-	-	-	-	-	-
All Bacterial^a^	81 (79–83)	-	951/31	-	-	-
*Burkholderia pseudomallei*	90 (85–93)	**0.010**	45/2	-	-	-
*Escherichia coli*	84 (79–89)	0.972	64/7	-	-	-
*Staphylococcus aureus*	83 (79–87)	0.972	118/8	-	-	-
*Staphylococcus*, other	91 (88–94)	**< 0.001**	58/4	-	-	-
*Streptococcus pneumoniae*	83 (76–89)	0.972	39/6	-	-	-
*Streptococcus*, other	82 (75–88)	0.972	43/8	-	-	-
Intracellular bacteria	83 (79–88)	0.178	100/4	-	-	-
Extracellular bacteria	84 (81–86)	0.173	415/17	-	-	-
All Viral^a^	-	-	-	82 (80–83)	-	1679/37
Adenovirus	-	-	-	74 (62–84)	**0.030**	30/2
Enterovirus	-	-	-	84 (76–89)	0.937	58/3
Influenza	-	-	-	89 (87–90)	**< 0.001**	431/19
Rhinovirus	-	-	-	74 (70–78)	**< 0.001**	209/9
RSV	-	-	-	81 (79–84)	0.083	406/13
**Age**	-	-	-	-	-	-
Adult^a^	82 (80–83)	-	1183/18	88 (86–89)	-	1268/14
12–18 years	82 (78–85)	0.299	132/6	88 (84–92)	0.631	95/6
2–11 years	70 (67–73)	**< 0.001**	373/7	79 (76–82)	**< 0.001**	352/10
3 months–1 year	73 (69–77)	**< 0.001**	183/8	80 (78–82)	**< 0.001**	576/17
<3 months	85 (82–88)	**0.002**	320/8	81 (79–84)	**< 0.001**	547/16
**Race**	-	-	-	-	-	-
All Subjects^a^	77 (76–79)	-	1389/12	80 (78–81)	-	1157/12
Asian	84 (79–89)	**0.007**	87/9	84 (76–91)	0.277	33/7
Black	79 (75–82)	0.059	311/11	77 (73–81)	0.277	254/12
White	76 (73–78)	**0.028**	684/11	80 (78–82)	0.784	686/12
Other	71 (64–78)	**0.010**	72/5	74 (67-82)	0.277	79/6
**Ethnicity**	-	-	-	-	-	-
Not Hispanic or Latino^a^	75 (73–77)	-	407/4	79 (77–81)	**-**	474/5
Hispanic or Latino	80 (77–84)	**< 0.001**	302/9	85 (81–88)	**< 0.001**	220/11
**Severity**	-	-	-	-	-	-
Non-ICU^a^	73 (66–79)	-	43/2	83 (79–86)	-	117/3
ICU	69 (66–73)	0.279	182/8	86 (82–90)	0.105	107/7

Average accuracies and 95% confidence intervals of bacterial and viral classification, stratified by different clinical parameters. Only groups with at least fifteen subjects across at least two datasets were evaluated. *P*-values represent statistical significance, comparing the group to its reference population. *N* is represented by the number of subjects/the number of datasets used for validation. The “Intracellular Bacteria” group includes subjects with *B. pseudomallei*, *S. typhi*, and *Mycoplasma* infection*.* The “Extracellular Bacteria” group includes all other subjects with bacterial infection for which an identified pathogen was available. For comparisons related to “Race,” the “All Subjects” group represents all subjects for which racial information was available. ^a^ Indicates the reference population used for determination of significance

We next evaluated signature performance by race and ethnicity. There were no significant differences due to race for viral classification. In contrast, there were racial differences in bacterial classification. Accuracies were higher for Asian subjects (84%, 95% CI 79–89%), they were similar to the population average for Black subjects (79%, 95% CI 75–82%), and they were lower for White subjects and other subjects (76%, 95% CI 73–78% and 71%, 64–78%, respectively). We also found that accuracies were higher in Hispanic subjects for both bacterial and viral classification as compared to non-Hispanic subjects (*P* < 0.001 for both comparisons; Table 4). Finally, we evaluated the impact of critical illness as defined by ICU admission on the ability of these signatures to identify the infection etiology but observed no significant performance difference in either bacterial or viral classification (Table 4). PPV and NPV values were calculated for these various comparisons (Additional file 1: Table S4).

Certain phenotypes or subject characteristics may be prone to high rates of misclassification. To identify such scenarios, we examined subjects where >80% of signatures classified the subject incorrectly (*n*=39 bacterial, *n*=70 viral). We could not identify any specific patterns with respect to age, pathogen, phenotype, or infection severity.

### COVID-19

Though most of the 28 published gene signatures included in this analysis were developed prior to the COVID-19 pandemic, we evaluated their performance in thirteen recently published COVID-19 datasets. Focusing on viral vs. non-viral classification, host gene expression signatures classified subjects with COVID-19 as having a viral infection with a median AUC of 0.85, after weighting for dataset size (Additional file 1: Table S5).

Four of the thirteen datasets included subjects with non-COVID viral and bacterial infections. Within these datasets, we evaluated each of the twenty-eight signatures’ ability to differentiate COVID-19 from the other phenotypes (including non-COVID-19 viral infections, bacterial infections, and healthy subjects). This COVID vs. non-COVID classifier performed well across signatures, with a median AUC of 0.80, after weighting for dataset size (Additional file 1: Table S5). Signature-specific performance is presented in Additional file 7: Table S10 and Additional file 8: Table S11.

## Discussion

In recent years, host gene expression has emerged as a promising diagnostic method to identify the etiology of suspected infectious diseases. However, to our knowledge, there has been no systematic comparison of these published host gene expression signatures. In this study, we evaluated 28 published gene expression signatures for their ability to differentiate bacterial from non-bacterial disease and viral from non-viral disease. The validation cohort consisted of 47 studies, 51 datasets, and 4589 subjects. Most significantly, we found that performance improved with larger signatures, viral classification was easier than bacterial classification, and that performance was decreased in pediatric subjects.

It is generally accepted that gene expression signatures should be applied to the same populations as they were derived from [43]. The signatures included here derived from studies that varied in subject age, infection etiologies, illness severity, and other unreported variables. However, we showed that signatures, particularly larger ones, can be retrained to classify more heterogeneous populations. Larger gene signatures encompass a larger swath of the relevant biology, are more adaptable, and therefore can be better tuned to a more diverse set of patient populations. This was consistent with our finding that performance increased with the number of genes in a signature.

This study compared each signature’s performance to the others, but this should not imply that any one signature is the best to use for clinical applications. For example, one might advocate for the largest and most accurate signature. Practical considerations might arise in measuring such signatures, although technologies continually evolve, enabling an ever-increasing degree of multiplexing while still offering rapid and precise measurements. For example, the BioFire system has been used to measure up to 45 host gene expression targets in about 45 min [44]. There are other considerations in choosing a “best” signature. The pre-test probability of bacterial infection will impact the utility of any given signature and how many false positives and false negatives one might expect. Patient characteristics are also important. For example, a mildly ill outpatient could tolerate a higher error rate since many are already managed with a “watch and wait” strategy. In this scenario, a NPV of 89% (the average NPV for bacterial classification) might be sufficient to withhold treatment. In contrast, the threshold to treat critically ill patients with antibacterials is lower. Here, a NPV of 89% may be inadequate while a PPV of 65% (average PPV for bacterial classification) is likely sufficient to start antibacterial therapy in an untreated patient. Patient and provider preferences are also relevant parameters in deciding whether a particular signature is sufficiently accurate to be clinically useful. Among the next steps is a real-world implementation study assessing a signature’s utility in the full spectrum of clinical illness. Doing so would also inform how the test might help patients whose diagnosis remains indeterminate despite adjudication.

While we found significant performance differences between individual gene signatures, we also identified patient characteristics that impacted classification accuracy. These performance differences were most pronounced when comparing pediatric and adult subjects where accuracy was lower in children, specifically those <12 years. Based on the available data, it is not possible to explain this lower performance in pediatric subjects. It is unlikely to be due to an outlier study since the effect was observed across multiple studies. One possibility is that children are known to have high rates of asymptomatic viral shedding compared to adults [45]. Consequently, children may have been classified as viral based on carriage when in fact, they had a non-viral etiology. In this scenario, the error would be in the clinically adjudicated phenotype rather than gene expression misclassification.

Our analyses also showed pathogen-related differences. These pathogen-specific performance differences may be caused by biological differences in the host immune response. The host response relies on the activation of multiple pathogen recognition receptors, each associated with a different type of pathogen [46]. Thus, some pathogens induce variable or alternative host transcriptional responses. The observed pathogen differences could also be due to differential rates of incorrect clinical adjudications. Subjects adjudicated as having a rhinovirus infection, which is a frequent colonizing microbe, may actually have had non-viral infections leading to errors in the clinical label [45]. Finally, the more severe infections associated with influenza and *B. pseudomallei* may create a more pronounced host response that is easier to detect. We were unable to control for severity of illness within pathogen groups since this information was not available in most cases. Whereas we were unable to identify a relationship between pathogen and severity, we found that bacterial/viral discrimination was similar in critically ill patients as it was in a less ill population. This is consistent with multiple signatures developed to distinguish sepsis from SIRS [13, 14, 21, 24].

We observed performance differences due to race and ethnicity that were consistent with ancestry-based variation in the immune response [47, 48]. However, they could also be due to other confounding variables including dataset-specific factors, such as clinical adjudication accuracy. Future studies should aim to enroll a heterogeneous population with stratification by race and ethnicity to support ongoing inquiry into this question.

In addition to subject-specific characteristics, we also identified analytical and technical variables that impacted performance. Signature performance was significantly decreased when discriminating more than two phenotypes (e.g., bacterial, viral, and SIRS) compared to only two phenotypes. This is likely due to the high biological variation in those datasets. However, the lower accuracy in classifying more complex scenarios needs to be weighed against the greater clinical utility such tests may offer. For example, discriminating bacterial, viral, and non-infectious illness simultaneously is more clinically useful than focusing only on bacterial or viral infection. The latter case requires an a priori assumption that an infection is present. This is reasonable in many cases but does limit generalizability. We also observed higher accuracies when validating in datasets derived from peripheral whole blood as compared to PBMCs. This difference could be explained by additional transcriptional information included in those cells that are removed during PBMC processing or perhaps changes in transcription that occur during PBMC processing [49, 50].

Finally, we showed that host gene expression classifiers developed prior to the SARS-CoV-2 pandemic correctly identified COVID-19 infections as viral. Furthermore, the same signatures could be trained to discriminate COVID-19 from other viral and bacterial infections, similar to published COVID-specific signatures [51]. Such signatures could be utilized as a supplement to RT-PCR testing to increase the clinical sensitivity of COVID-19 detection [52].

Overall, our analyses were limited by the accuracy and breadth of annotations provided in the publicly available validation datasets. Focusing only on subjects with a definitive diagnosis excludes a large number of people with an indeterminate diagnosis. Though there is no evidence that host response would differ in these subjects, the signatures’ accuracies cannot be accurately assessed in the absence of a reliable reference standard in these cases. The significant heterogeneity between validation studies may have also limited our findings. We assumed that this heterogeneity would “average out” after pooling patients together; however, this may not have been the case. Future studies validating host gene expression signatures should consider stratifying by the variables we showed to impact test performance. It is important to note that the original studies that described the 28 evaluated signatures often used other methodologies for their validation experiments. For example, some signatures rely on the calculation of a “diagnostic score” which is then used for subject classification. Because of the differences in methodology, we do not expect our results to perfectly match those in the original publications. Additionally, we did not seek to maximize the performance of any given signature. Rather, we chose one method for processing and validation that could be applied equally to all signatures. This allowed us to make comparisons within the context of this study.

## Conclusions

This analysis validated 28 previously described host gene expression signatures in a common validation cohort comprised of 4589 subjects in addition to 1416 subjects in thirteen COVID-19-related datasets. These signatures have not been systematically reviewed and compared to one another. To our knowledge, this type of summary review of published gene expression signatures has not been performed for any other clinical application. With such an analysis, we found that the performance of published host gene expression signatures does not differ dramatically, underscoring that many signatures may exist to answer the same clinical question. However, we found that signature performance differed based on age, specific pathogen, sample type, and the cohort’s heterogeneity. It is unlikely that these performance differences could have been discovered in a single validation cohort or when evaluating just one signature. These findings will be critical for the development and translation of host gene expression signatures into clinical practice.

## Supplementary Information

**Additional file 1:.** Document containing supplementary tables S1-S5 and supplementary figures S1-S3

**Additional file 2: Table S6.** Standardized Annotations of Subjects.

**Additional file 3: Table S7.** Gene List of Evaluated Signatures.

**Additional file 4.** Cross-Validation Code (.py). Code written in Python used to run leave-one-out cross-validation on public gene expression data. This file can be viewed with most text editing applications, including TextEdit on Mac and Windows Notepad on Windows.

**Additional file 5: Table S8.** Gene Signature Characteristics (.xlsx).

**Additional file 6: Table S9.** Validation Dataset Characteristics.

**Additional file 7: Table S10.** Signature-Specific Performance in COVID-19 Classification.

**Additional file 8: Table S11.** Heterogeneity in DOR of COVID-19 Classification Signatures.

## Data Availability

The analyses performed in this study are based on publicly available datasets. These data can be found in Gene Expression Omnibus, ArrayExpress, or the Genome Sequencing Archive using the specified accession numbers found in Additional file 6: Table S9. Standardized annotations of the subjects in this study can be found in Additional file 2: Table S6. The python code used to run leave-one-out cross-validation on public gene expression data can be found in Additional file 4.
