# Supplementary material for: Systematic comparison of published host gene expression signatures for bacterial/viral discrimination
Source: Genome Med. 2022 Feb 21;14:18. doi: 10.1186/s13073-022-01025-x (PMC8858657; doi:10.1186/s13073-022-01025-x)

# Supplementary Tables and Figures for:

**Title: Systematic Comparison of Published Host Gene Expression Signatures for Bacterial/Viral Discrimination**

**Authors:** Nicholas Bodkin, Melissa Ross, Micah T. McClain, Emily R. Ko, Christopher W. Woods, Geoffrey S. Ginsburg, Ricardo Henao, Ephraim L. Tsalik

**List of Supplementary Materials:**

**Table S1.** Conditions Represented in Validation Cohort.

**Table S2.** Top 20 Genes in Composite Signature by Average Coefficient.

**Table S3.** Heterogeneity in DOR of Bacterial and Viral Classification Signatures.

**Table S4.** Predictive Values in Patient Subgroups.

**Table S5.** Overall Signature Performance in COVID-19 Classification.

**Fig. S1.** Flow Diagram for the Inclusion of Validation Datasets.

**Fig. S2.** HSROC Curves for Evaluated Signatures.

**Fig. S3.** Signature Performance by Validation Dataset.

**Table S1. Conditions represented in validation datasets**.

| Condition | Number of cases |
|---|---|
| All Viral Infections | 1679 |
| Viral infection, NOS | 451 |
| Influenza | 431 |
| Respiratory Syncytial Virus | 406 |
| Rhinovirus | 209 |
| Enterovirus | 58 |
| Poly-viral | 66 |
| Adenovirus | 31 |
| Human Herpesvirus 6 | 10 |
| Other | 12 |
| | |
| All Bacterial Infections | 951 |
| Bacterial Infection, NOS | 469 |
| *Staphylococcus aureus* | 118 |
| *Escherichia coli* | 64 |
| *Burkholderia pseudomallei* | 45 |
| Unspecified *Staphylococcus* | 40 |
| *Streptococcus pneumoniae* | 39 |
| *Mycoplasma* | 30 |
| *Salmonella typhi* | 25 |
| Coagulase-Negative *Staphylococcus* | 16 |
| *Streptococcus pyogenes* | 14 |
| Unspecified *Streptococcus* | 12 |
| Poly-bacterial | 11 |
| Other | 68 |
| | |
| All Non-Infectious Illnesses | 537 |
| SIRS, NOS | 306 |
| Systemic Lupus Erythematosus | 110 |
| Kawasaki Disease | 90 |
| Still's Disease | 31 |
| | |
| Healthy | 1427 |

Validation datasets include patients with a wide range of conditions. Species with less than 10 subjects are grouped into "Other". NOS = Not Otherwise Specified. SIRS = Systemic Inflammatory Response Syndrome.

**Table S2. Top 20 Genes in Composite Signature by Average Coefficient.**

| Rank | Bacterial vs. non-Bacterial | | | Viral vs. non-Viral | | |
|---|---|---|---|---|---|---|
| | **Gene Name** | **Ensembl ID** | **Avg. Coefficient** | **Gene Name** | **Ensembl ID** | **Avg. Coefficient** |
| 1 | CETP | ENSG00000087237 | 0.0217 | IFI27 | ENSG00000165949 | 0.0702 |
| 2 | RPGRIP1 | ENSG00000092200 | -0.0181 | OTOF | ENSG00000115155 | 0.0190 |
| 3 | FCER1A | ENSG00000179639 | -0.0167 | FCER1A | ENSG00000179639 | -0.0188 |
| 4 | IFI27 | ENSG00000165949 | -0.0165 | LARP1 | ENSG00000155506 | 0.0160 |
| 5 | PDE9A | ENSG00000160191 | -0.0151 | OAS1 | ENSG00000089127 | 0.0155 |
| 6 | PLAC8 | ENSG00000145287 | 0.0146 | XAF1 | ENSG00000132530 | 0.0140 |
| 7 | SLPI | ENSG00000124107 | 0.0126 | IRF9 | ENSG00000213928 | 0.0140 |
| 8 | JUP | ENSG00000173801 | -0.0126 | KREMEN1 | ENSG00000183762 | 0.0138 |
| 9 | ADGRE1 | ENSG00000174837 | 0.0125 | QARS | ENSG00000172053 | -0.0134 |
| 10 | ZNF823 | ENSG00000197933 | -0.0118 | IFI44 | ENSG00000137965 | 0.0132 |
| 11 | LILRB1 | ENSG00000104972 | 0.0115 | AL136295.5 | ENSG00000259529 | 0.0132 |
| 12 | NRG1 | ENSG00000157168 | 0.0113 | KLRB1 | ENSG00000111796 | -0.0132 |
| 13 | VPS13A | ENSG00000197969 | 0.0113 | ADGRE3 | ENSG00000131355 | -0.0131 |
| 14 | LTA4H | ENSG00000111144 | 0.0110 | RSAD2 | ENSG00000134321 | 0.0130 |
| 15 | VAMP5 | ENSG00000168899 | 0.0109 | EEF1G | ENSG00000254772 | -0.0125 |
| 16 | YWHAE | ENSG00000108953 | 0.0109 | LY6E | ENSG00000160932 | 0.0120 |
| 17 | ACTR2 | ENSG00000138071 | 0.0107 | EEF1B2 | ENSG00000114942 | -0.0119 |
| 18 | TSPO | ENSG00000100300 | 0.0107 | EIF4B | ENSG00000063046 | -0.0117 |
| 19 | ANKRD20A11P | ENSG00000215559 | -0.0106 | AC000120.1 | ENSG00000243107 | -0.0116 |
| 20 | ADK | ENSG00000156110 | -0.0105 | AP002990.1 | ENSG00000255508 | -0.0109 |

Relative gene importance was characterized by the average of each gene's coefficient in all models. For genes that mapped to multiple microarray probes, the coefficient with the largest magnitude was used for the average. This analysis was performed using coefficient data from the composite signature comprised of 864 genes.

**Table S3. Heterogeneity in DOR of Bacterial and Viral Classification Signatures.**

| Signature | Bacterial vs. non-Bacterial | | | Viral vs. non-Viral | | |
|---|---|---|---|---|---|---|
| | % Heterogeneity (95% CI) | Q-statistic | p-value | % Heterogeneity (95% CI) | Q-statistic | p-value |
| TS1 | 52 [27.1-68.4] | 60.40 | < 0.001 | 79.4 [72.3-84.7] | 179.89 | < 0.001 |
| HL2 | 66.2 [51.3-76.5] | 94.59 | < 0.001 | 67.2 [54.3-76.5] | 116.02 | < 0.001 |
| LC2 | 53.8 [30.1-69.5] | 62.79 | < 0.001 | 50.6 [26.6-66.7] | 66.75 | < 0.001 |
| XW2 | 46.7 [19.8-64.5] | 60.00 | 0.002 | 66.4 [53.2-75.9] | 116.01 | < 0.001 |
| GS3 | 72.7 [59.6-81.5] | 91.46 | < 0.001 | 66.1 [51.2-76.5] | 94.49 | < 0.001 |
| LS3 | 61.6 [44-73.7] | 83.35 | < 0.001 | 67 [54-76.4] | 115.19 | < 0.001 |
| SB4 | 70.1 [55.1-80.1] | 80.36 | < 0.001 | 57.4 [36.9-71.2] | 72.76 | < 0.001 |
| SK7 | 67.6 [51.3-78.4] | 77.11 | < 0.001 | 71.5 [59.6-79.9] | 112.32 | < 0.001 |
| SB8 | 72.5 [58.7-81.7] | 83.55 | < 0.001 | 61.7 [43.8-73.9] | 80.91 | < 0.001 |
| RC10 | 74.1 [62.3-82.2] | 100.40 | < 0.001 | 71.9 [60.7-80] | 121.10 | < 0.001 |
| SN10 | 70 [54.6-80.2] | 76.76 | < 0.001 | 66 [50.7-76.6] | 91.21 | < 0.001 |
| SR10 | 69.9 [56.8-79] | 102.86 | < 0.001 | 70.3 [58.9-78.5] | 127.95 | < 0.001 |
| AK11 | 68.2 [53.7-78.2] | 91.30 | < 0.001 | 73.2 [62.6-80.8] | 126.96 | < 0.001 |
| BF11 | 67.1 [52.7-77.1] | 97.24 | < 0.001 | 51.4 [30.2-66.2] | 80.30 | < 0.001 |
| NC19 | 63.7 [47.3-74.9] | 88.05 | < 0.001 | 70.9 [60-78.8] | 134.09 | < 0.001 |
| SL20 | 67.7 [52.8-77.9] | 89.80 | < 0.001 | 61 [43.9-72.8] | 89.67 | < 0.001 |
| MW23 | 66.8 [51.9-77] | 93.25 | < 0.001 | 77.4 [69.6-83.2] | 172.66 | < 0.001 |
| ZG25 | 71.9 [60.1-80.1] | 113.70 | < 0.001 | 71.4 [60.6-79.3] | 133.06 | < 0.001 |
| MS29 | 71.8 [57.1-81.4] | 77.89 | < 0.001 | 75.9 [66.2-82.8] | 128.58 | < 0.001 |
| PT29 | 69.8 [56.5-79.1] | 99.36 | < 0.001 | 65 [50.9-75.1] | 108.67 | < 0.001 |
| RC31 | 73.9 [62.8-81.6] | 114.74 | < 0.001 | 62.9 [47.5-73.8] | 99.79 | < 0.001 |
| HS33 | 74.2 [63.4-81.9] | 116.50 | < 0.001 | 70.5 [59.2-78.7] | 128.82 | < 0.001 |
| HL34 | 72.5 [61.1-80.5] | 116.32 | < 0.001 | 77 [69-82.9] | 169.47 | < 0.001 |
| ZG48 | 63.9 [47.6-75.1] | 88.57 | < 0.001 | 70.7 [59.5-78.8] | 129.59 | < 0.001 |
| MR59 | 73.8 [63-81.5] | 118.51 | < 0.001 | 73.1 [63.2-80.4] | 141.52 | < 0.001 |
| TW96 | 70.5 [57.8-79.4] | 104.97 | < 0.001 | 77.5 [69.6-83.3] | 168.88 | < 0.001 |
| MW139 | 69.9 [57.1-78.9] | 106.39 | < 0.001 | 73.9 [64.4-80.8] | 149.16 | < 0.001 |
| AK398 | 73.3 [61.9-81.3] | 112.34 | < 0.001 | 77.2 [68.9-83.3] | 157.81 | < 0.001 |
| All | 66 [50.9-76.4] | 94.01 | < 0.001 | 70.4 [59.3-78.5] | 131.98 | < 0.001 |

Heterogeneity in the Diagnostic Odds Ratio (DOR) was evaluated for each host gene expression signature in bacterial and viral classification. Percent heterogeneity with a 95% confidence interval is presented with the Q-statistic and p-value. Values were computed using the Mantel-Haenszel method.

**Table S4. Predictive Values in Patient Subgroups.**

| Parameter | Bacterial vs. non-Bacterial | | | | Viral vs. non-Viral | | | |
|---|---|---|---|---|---|---|---|---|
| | PPV (%) | NPV (%) | Prevalence (%) | N (subjects/studies) | PPV (%) | NPV (%) | Prevalence (%) | N (subjects/studies) |
| **All subjects** | 65 (61-69) | 89 (87-91) | 32.7% | 2887 / 31 | 84 (81-86) | 84 (82-87) | 46.9% | 3584 / 37 |
| **Age** | - | - | - | - | - | - | - | - |
| Adult | 73 (68-79) | 88 (85-92) | 37.4% | 1183 / 18 | 85 (80-89) | 90 (87-93) | 41.4% | 1268 / 14 |
| 12 - 18 years | 61 (42-81) | 93 (84-100) | 26.3% | 132 / 6 | 80 (57-95) | 94 (84-100) | 35.6% | 95 / 6 |
| 2 - 11 years | 51 (41-61) | 87 (80-94) | 31.0% | 373 / 7 | 73 (62-83) | 82 (75-89) | 38.0% | 352 / 10 |
| 3 months - 1 year | 51 (36-66) | 89 (79-96) | 28.9% | 183 / 8 | 88 (83-93) | 70 (61-77) | 62.2% | 576 / 17 |
| <3 months | 84 (75-92) | 86 (79-93) | 44.2% | 320 / 8 | 90 (85-94) | 67 (58-76) | 68.7% | 547 / 16 |
| **Race** | - | - | - | - | - | - | - | - |
| All Subjects | 60 (54-66) | 90 (87-92) | 31.5% | 1389 / 12 | 81 (77-86) | 79 (74-83) | 49.3% | 1157 / 12 |
| Black | 70 (59-80) | 86 (78-93) | 41.1% | 311 / 11 | 78 (67-88) | 76 (66-85) | 50.2% | 254 / 12 |
| White | 52 (43-60) | 91 (88-95) | 26.1% | 684 / 11 | 82 (76-88) | 78 (72-83) | 50.6% | 686 / 12 |
| Asian | 81 (60-95) | 87 (70-100) | 42.7% | 87 / 9 | 69 (25-100) | 93 (75-100) | 29.5% | 33 / 7 |
| Other | 37 (9-64) | 91 (78-100) | 19.1% | 72 / 5 | 81 (58-100) | 69 (48-88) | 52.9% | 79 / 6 |
| **Ethnicity** | - | - | - | - | - | - | - | - |
| Hispanic or Latino | 68 (56-78) | 90 (83-96) | 34.6% | 302 / 9 | 85 (74-93) | 85 (76-93) | 46.0% | 220 / 11 |
| Not Hispanic or Latino | 56 (49-63) | 89 (85-92) | 30.3% | 407 / 4 | 81 (76-86) | 77 (71-82) | 50.7% | 474 / 5 |

Positive predictive values (PPV) and negative predictive values (NPV) with 95% confidence intervals of bacterial and viral classification, stratified by different clinical parameters. N is represented by the number of subjects / the number of datasets used for validation. The “All Subjects” group under the “Race” category represents all subjects for which racial information was available.

**Table S5. Overall Signature Performance in COVID-19 Classification.**

| Dataset Makeup | N | Viral vs. non-Viral | | COVID vs. non-Viral | | COVID vs. non-COVID | |
|---|---|---|---|---|---|---|---|
| | | Median AUC | IQR | Median AUC | IQR | Median AUC | IQR |
| **COVID + healthy** | 9 | 0.867 | [0.823-0.893] | - | - | - | - |
| **COVID + other infection + healthy** | 4 | 0.831 | [0.789-0.855] | 0.839 | [0.801-0.865] | 0.804 | [0.735-0.831] |

Viral vs. non-viral AUCs were calculated for each of the 29 signatures in thirteen COVID-19 datasets. The Viral vs. non-Viral metrics describe the ability of the signatures to classify COVID-19 as viral in nature when compared to healthy controls in nine datasets. These performance metrics can be compared to signature performance for viral classification more generally (Table 3). Four datasets included non-COVID infections such as other viral infections or bacterial infections. In this case, we report two additional performance measures. COVID vs. non-Viral describes the ability of the signatures to discriminate COVID-19 from other non-viral diseases (e.g., healthy or bacterial infection). The COVID vs. non-COVID comparison discriminated subjects with COVID-19 infection from all other phenotypes (other viral infections, bacterial infections, healthy). Mean AUCs were first generated for each signature across the datasets in the parameter group, weighted by the number of subjects in each validation dataset. The median of the weighted AUC values and IQR were then calculated and presented here. N represents the number of datasets for the specified cohort composition.

**Fig. S1. Flow Diagram for the Inclusion of Validation Datasets.** Transcriptome studies, consisting of microarray or RNA sequencing data, were systematically reviewed and selected from the Gene Expression Omnibus (GEO) and ArrayExpress with an approach similar to that outlined in the Preferred Reporting Items for Systematic Reviews and Meta-Analyses (PRISMA) statement. After screening the initial 781 studies, 47 studies met our inclusion criteria.

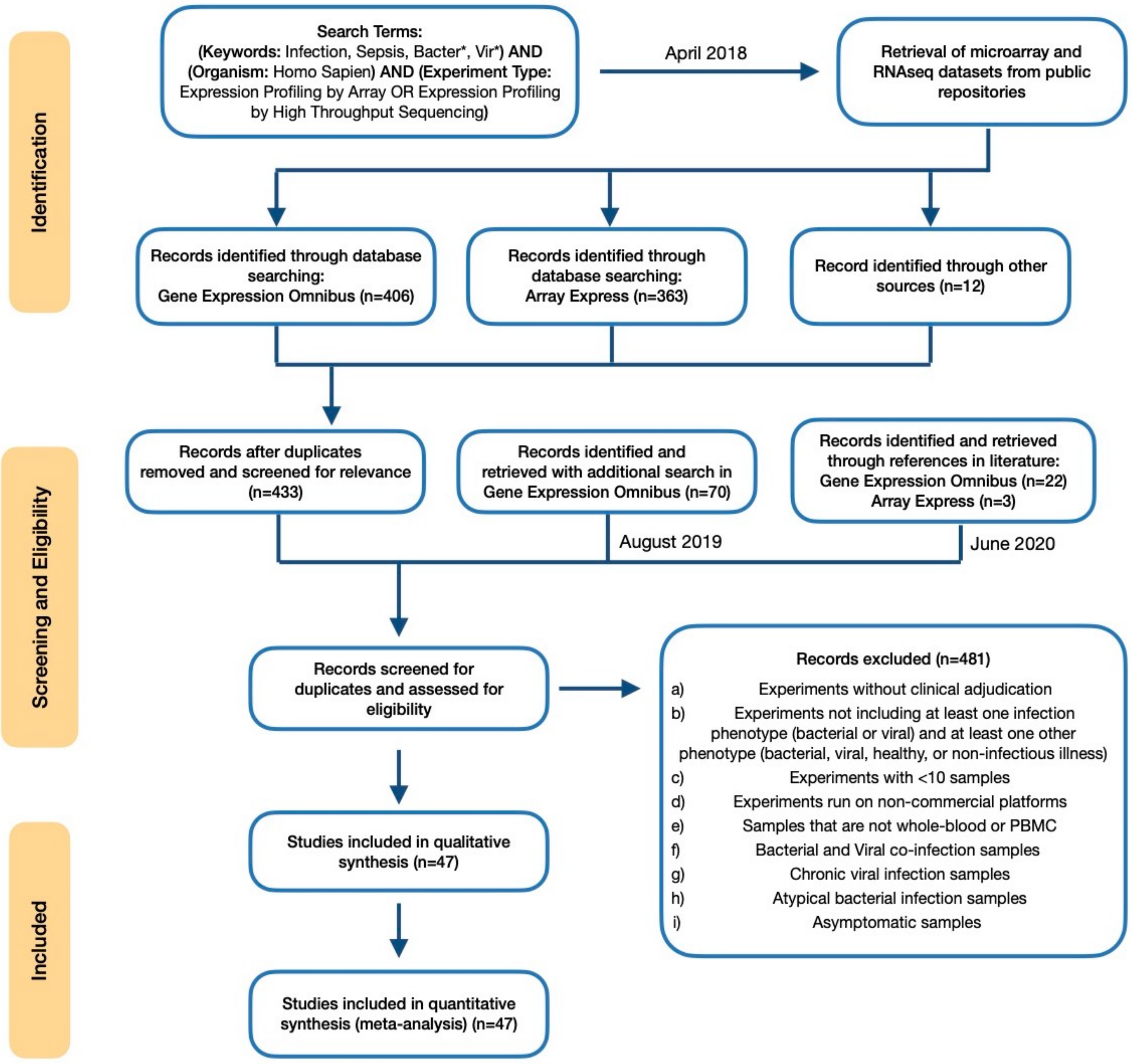

**Fig. S2. HSROC Curves for Evaluated Signatures.** Hierarchical summary ROC (HSROC) curves were generated for each signature, based on the signature's confusion matrices for all validation datasets. [1/14]

# TS1

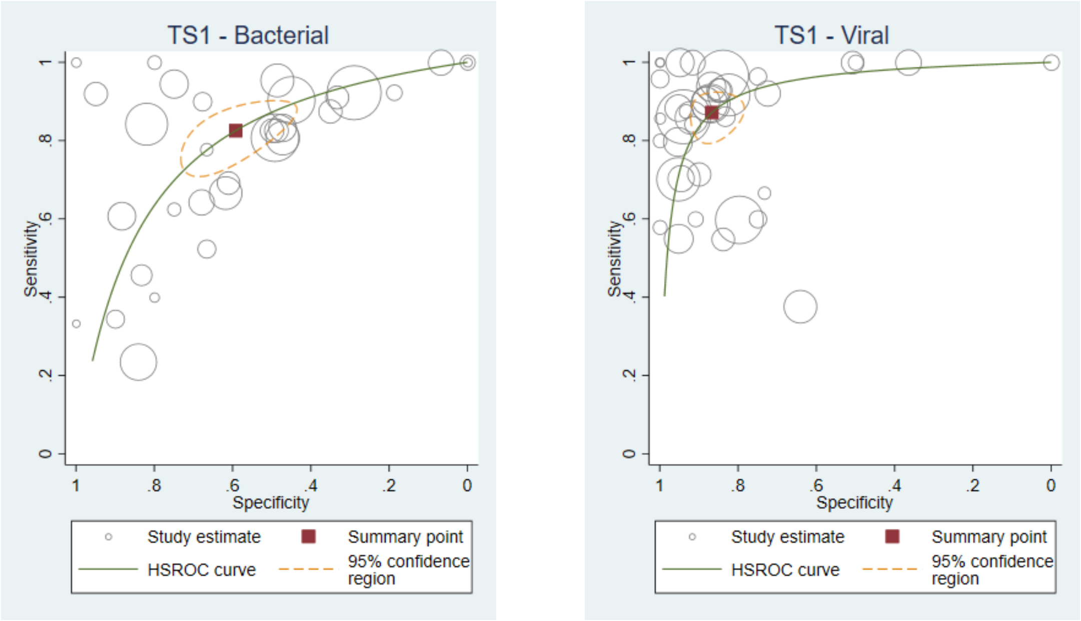


# HL2

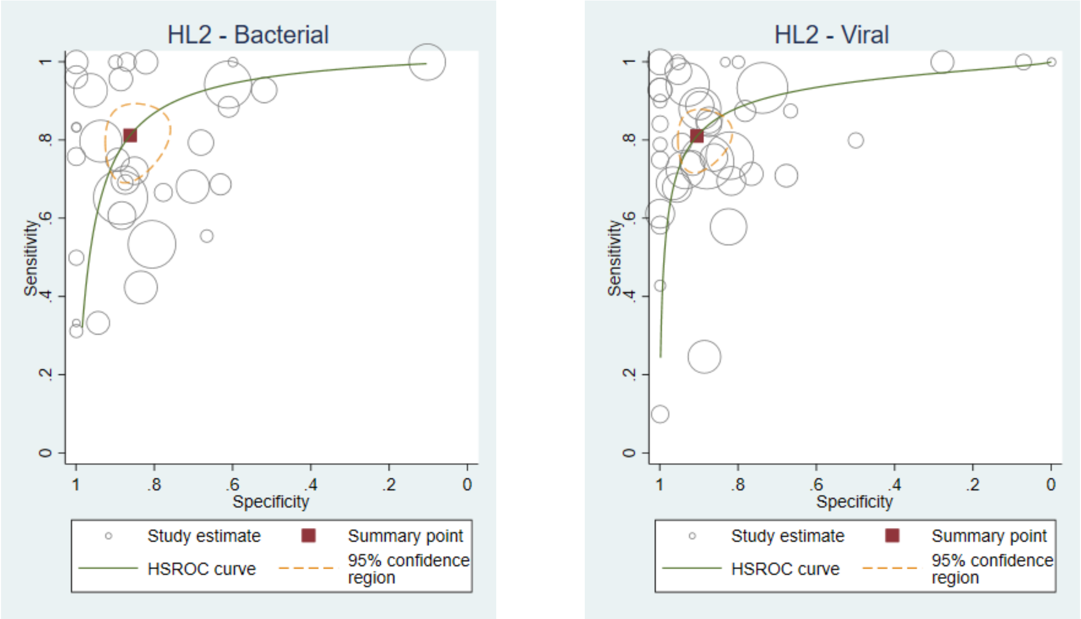

**Fig. S2. HSROC Curves for Evaluated Signatures (continued)** [2/14]

# LC2

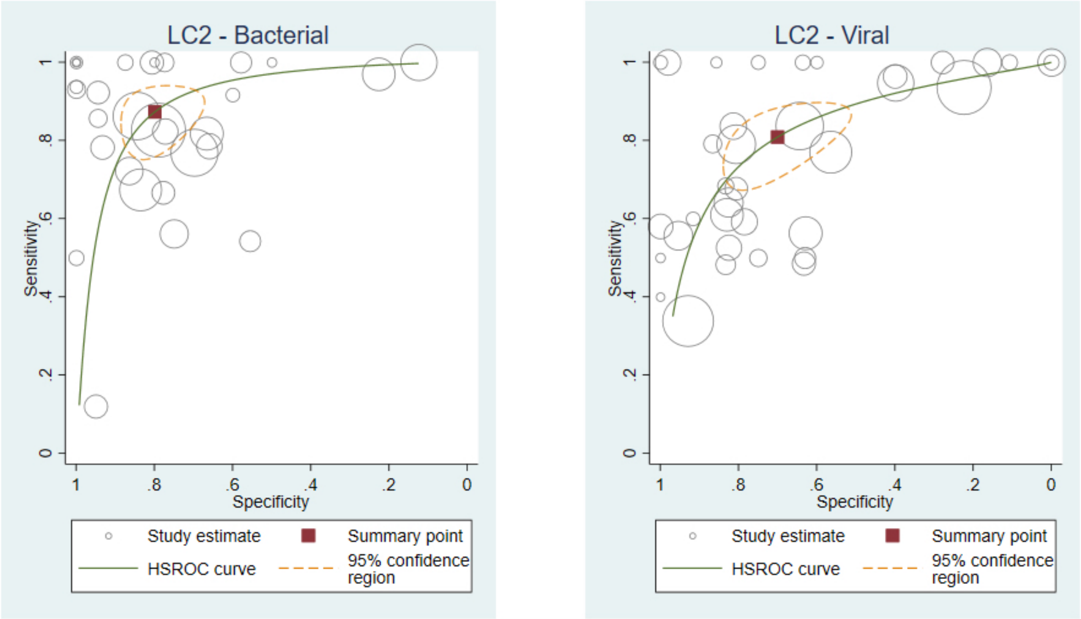


# XW2

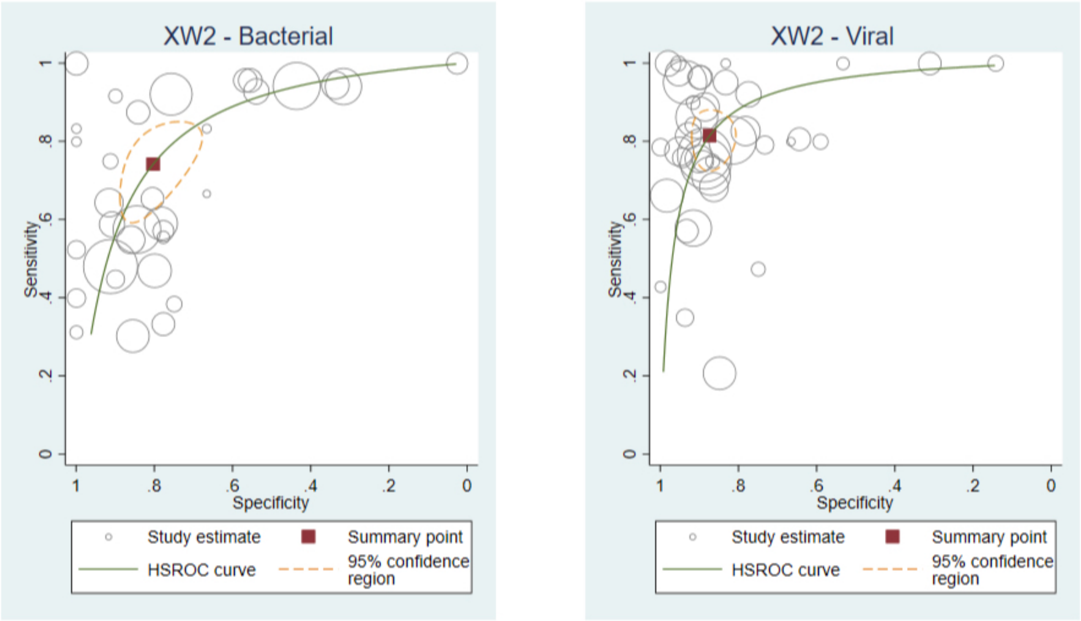

**Fig. S2. HSROC Curves for Evaluated Signatures (continued)** [3/14]

# GS3

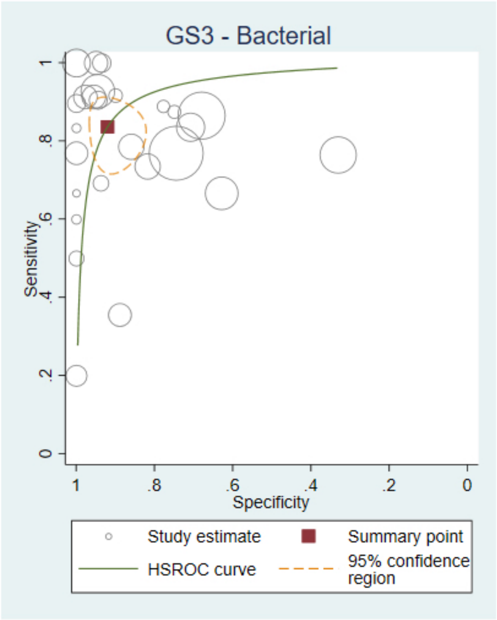


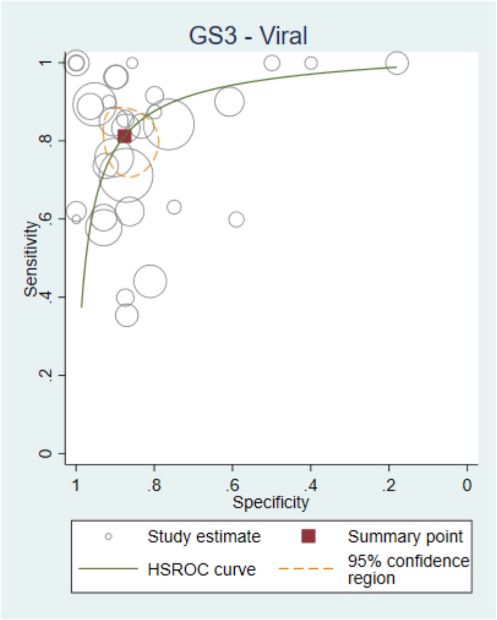


# LS3

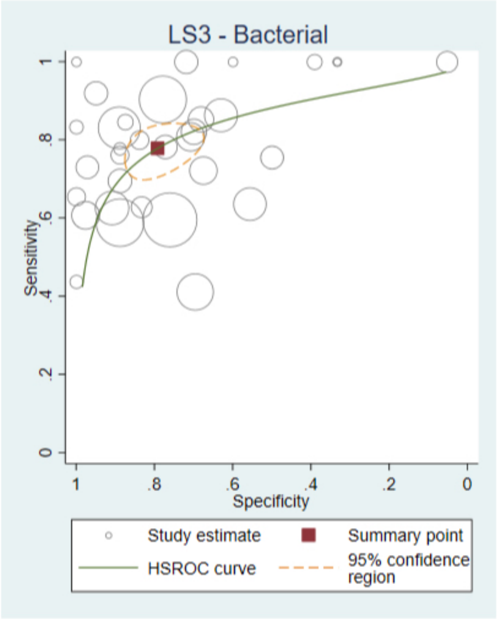


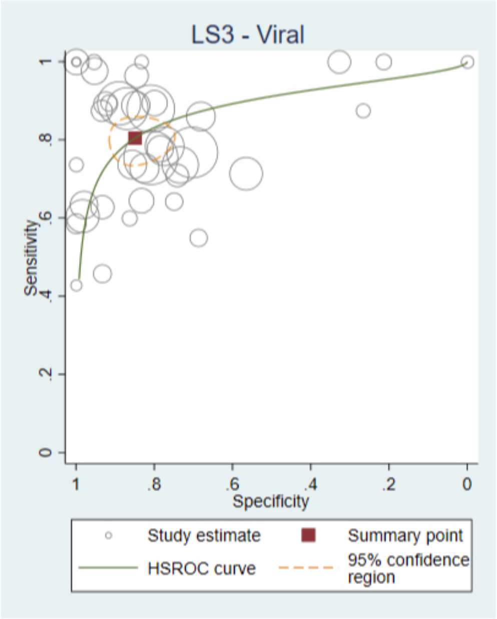

**Fig. S2. HSROC Curves for Evaluated Signatures (continued)** [4/14]

# SB4

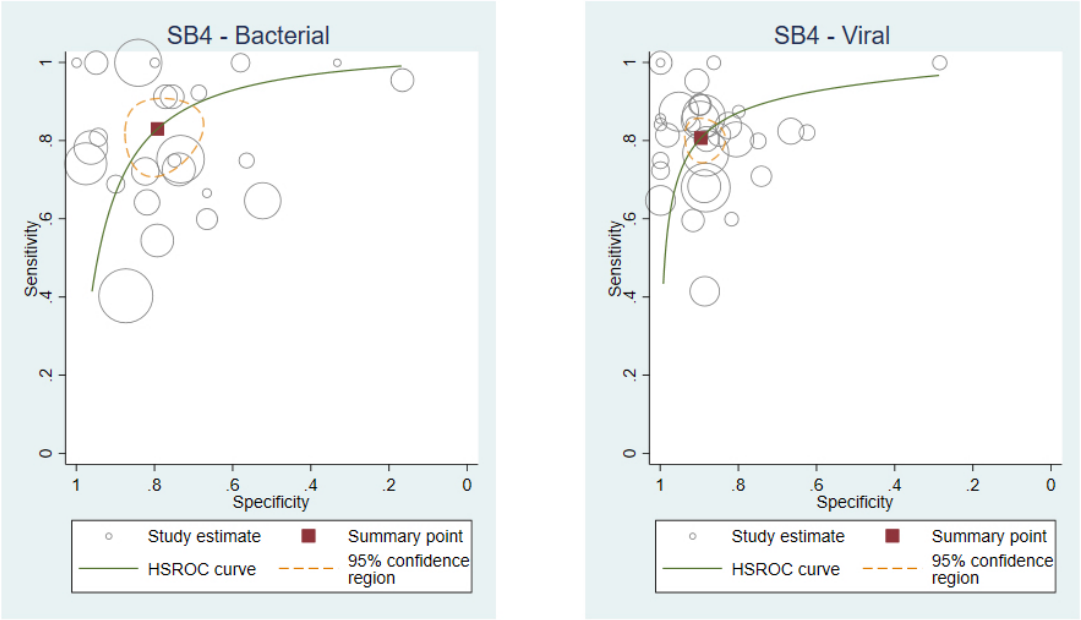


# SK7

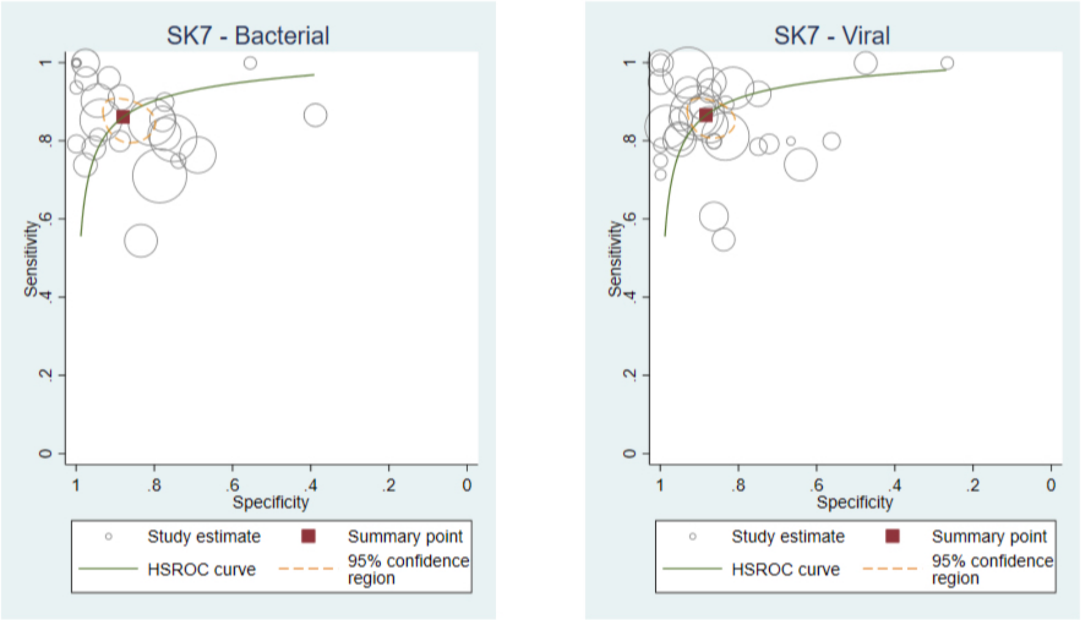

**Fig. S2. HSROC Curves for Evaluated Signatures (continued)** [5/14]

# SB8

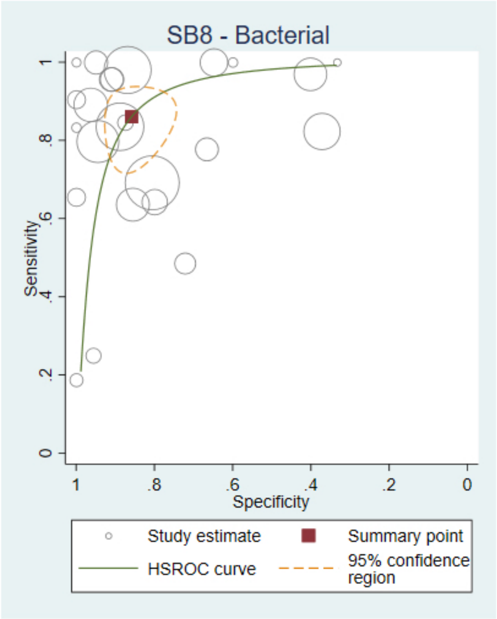


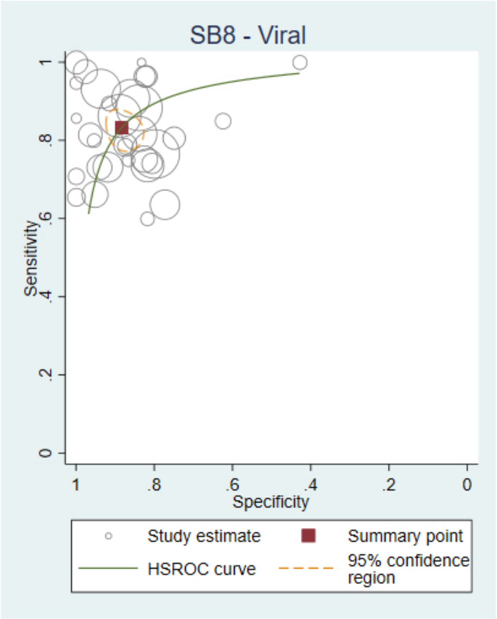


# RC10

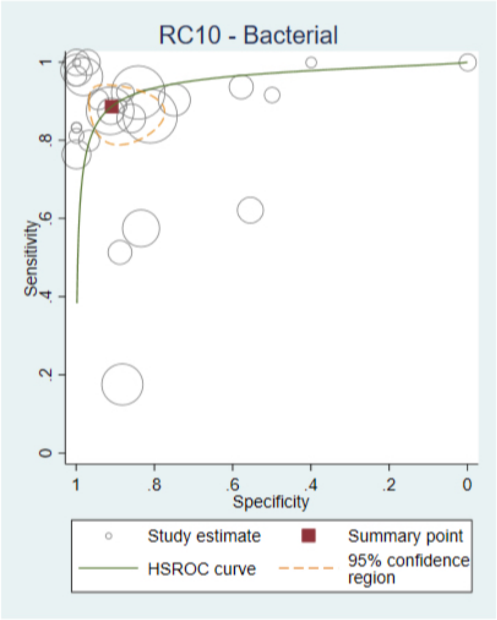


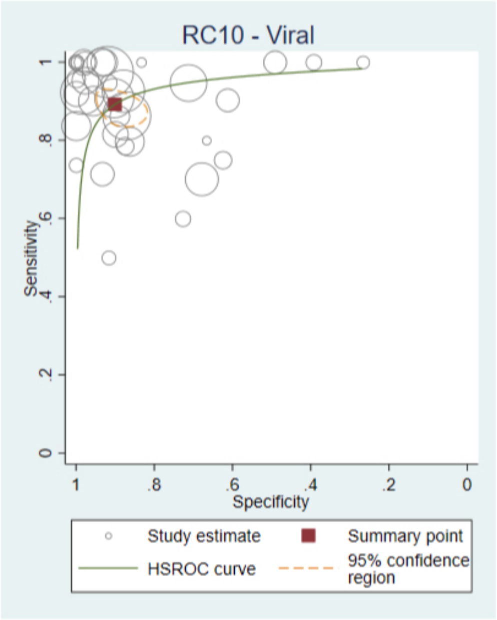

**Fig. S2. HSROC Curves for Evaluated Signatures (continued)** [6/14]

# SR10

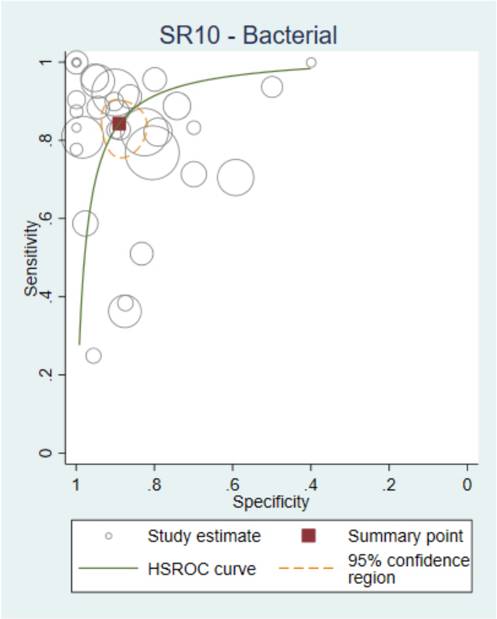


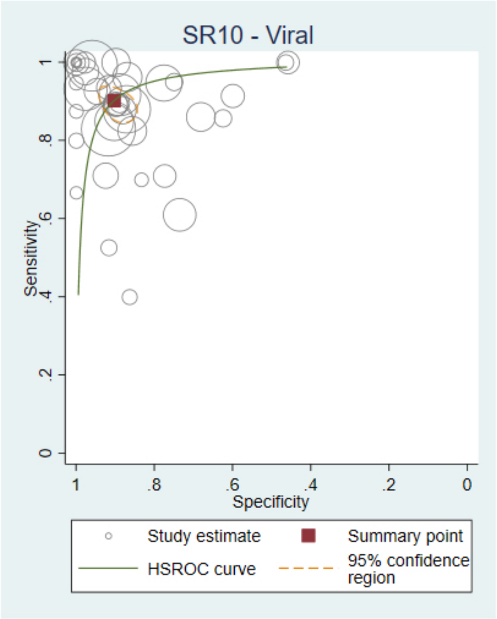


# SN10

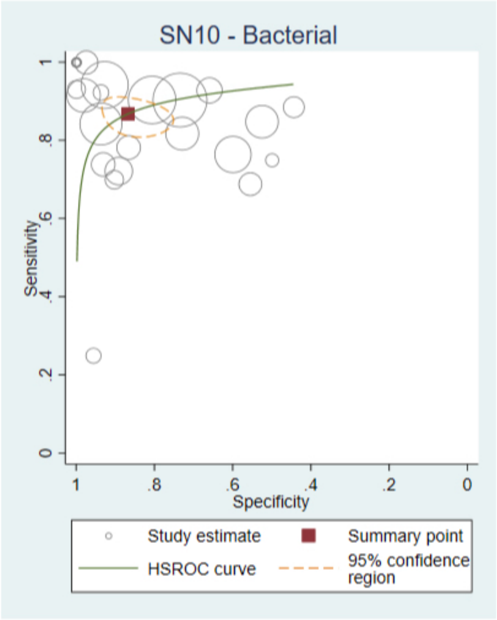


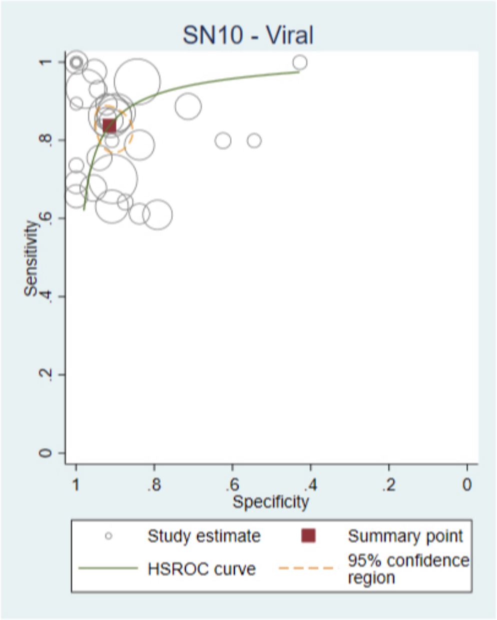

**Fig. S2. HSROC Curves for Evaluated Signatures (continued)** [7/14]

# AK11

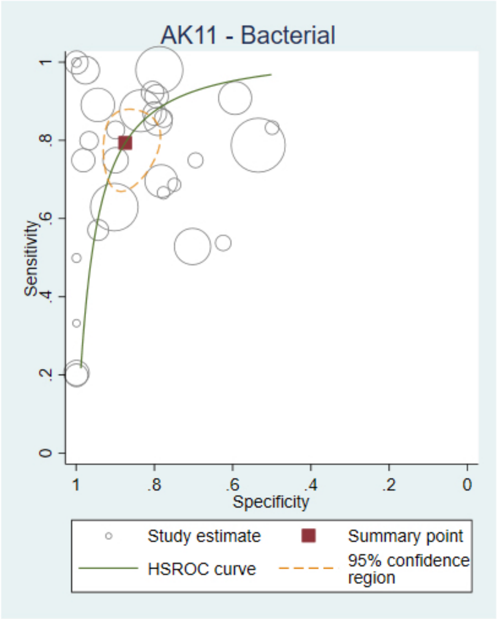


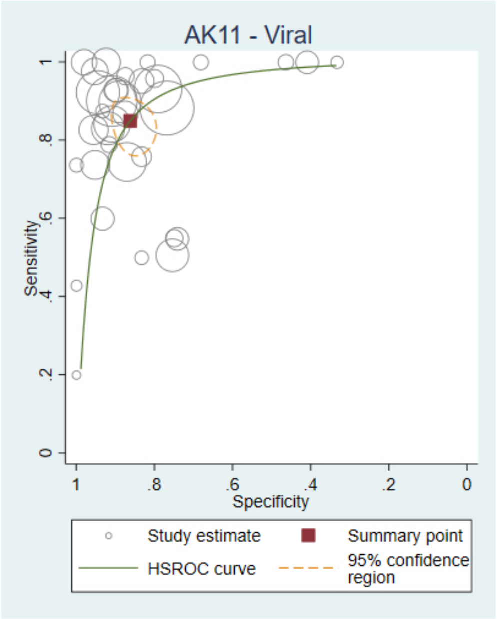


# BF11

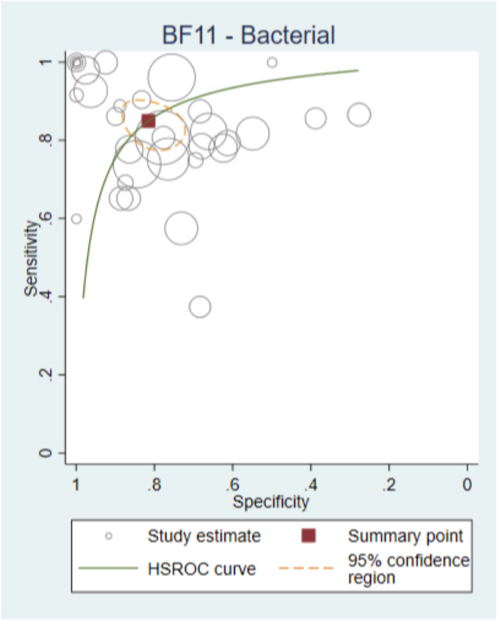


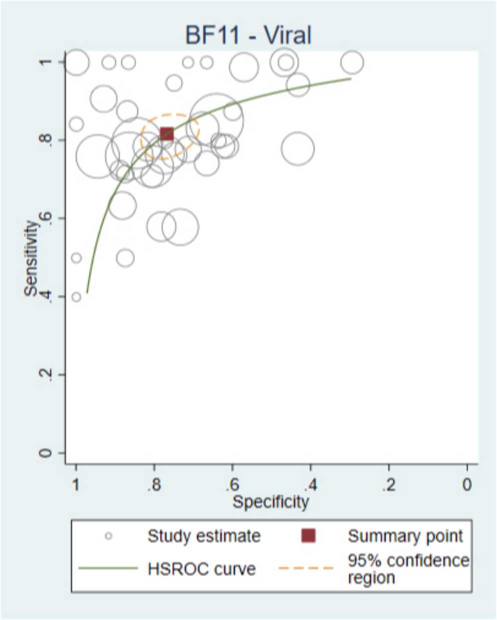

**Fig. S2. HSROC Curves for Evaluated Signatures (continued)** [8/14]

# NC19

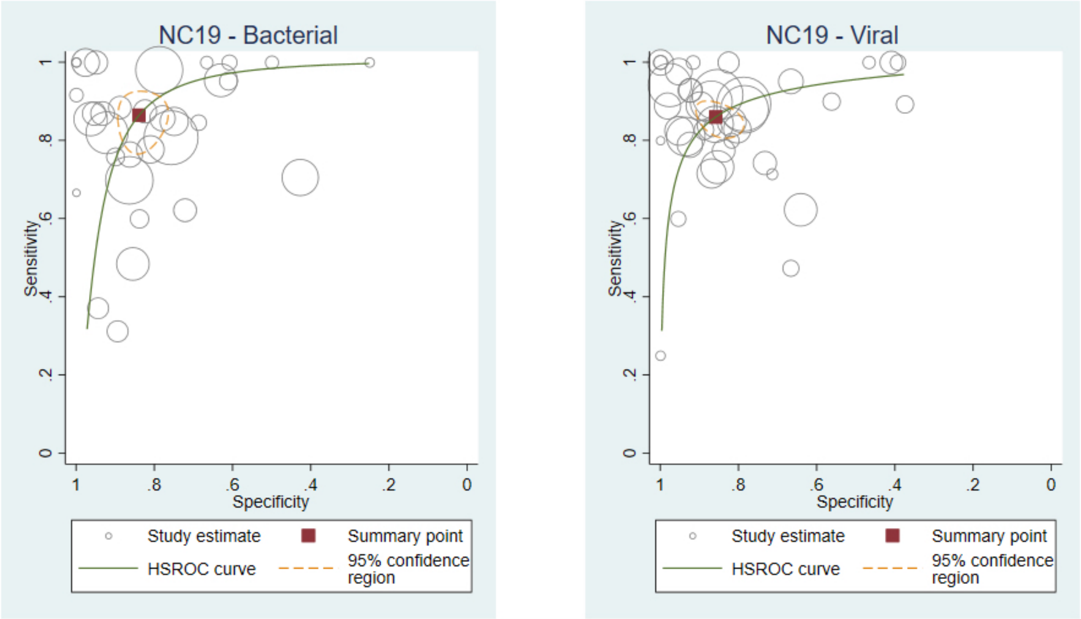


# SL20

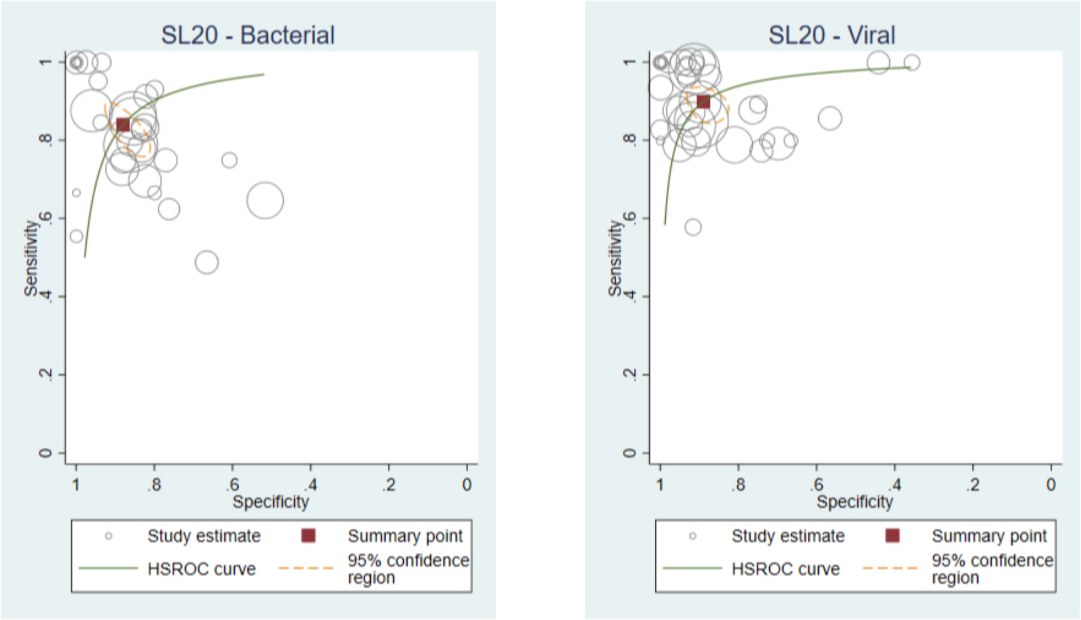

**Fig. S2. HSROC Curves for Evaluated Signatures (continued)** [9/14]

# MW23

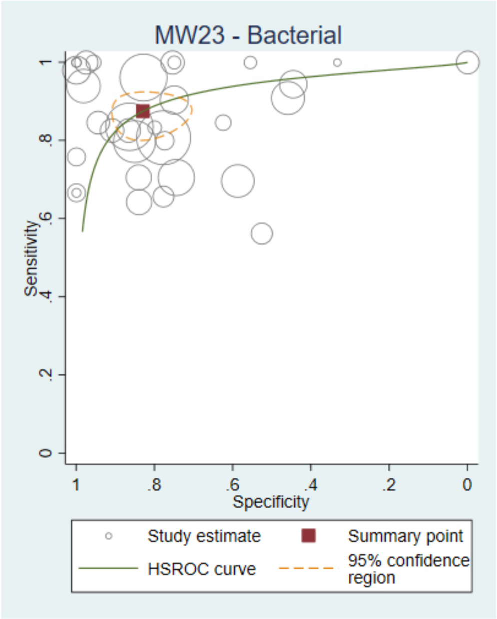


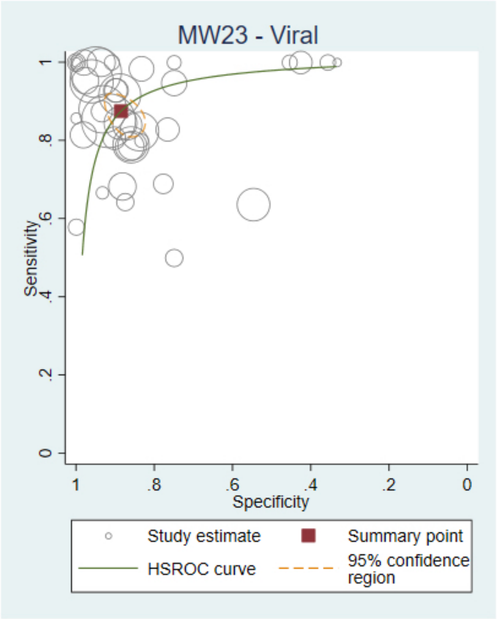


# ZG25

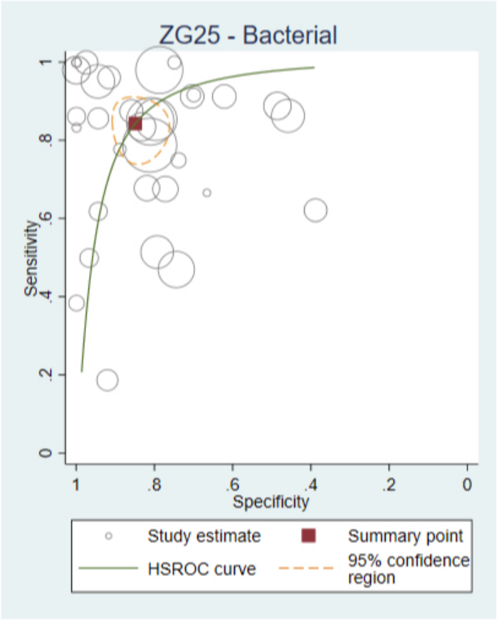


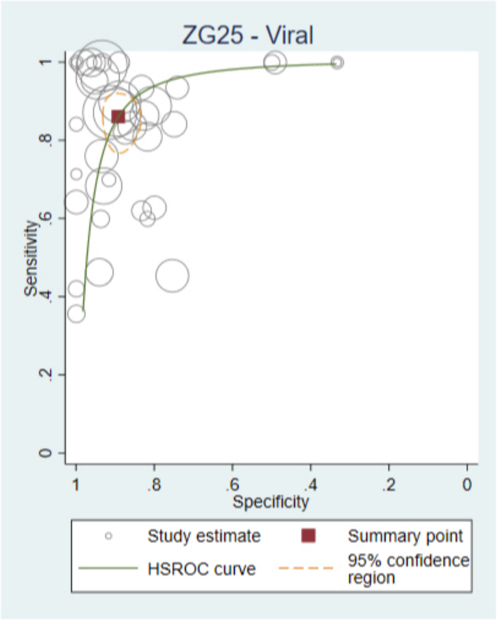

**Fig. S2. HSROC Curves for Evaluated Signatures (continued)** [10/14]

## MS29

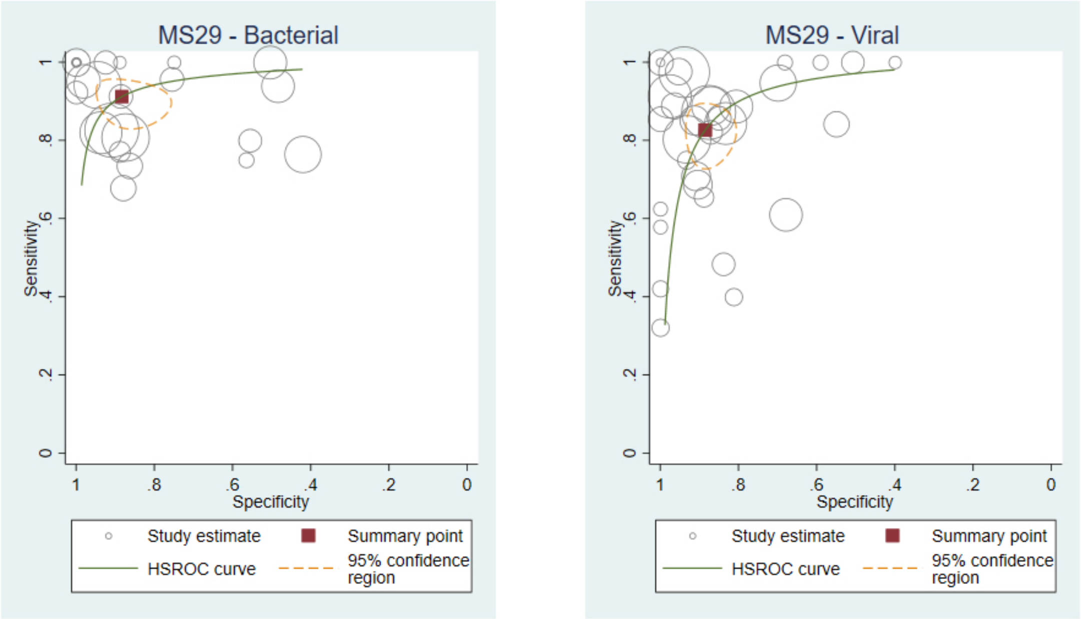


## PT29

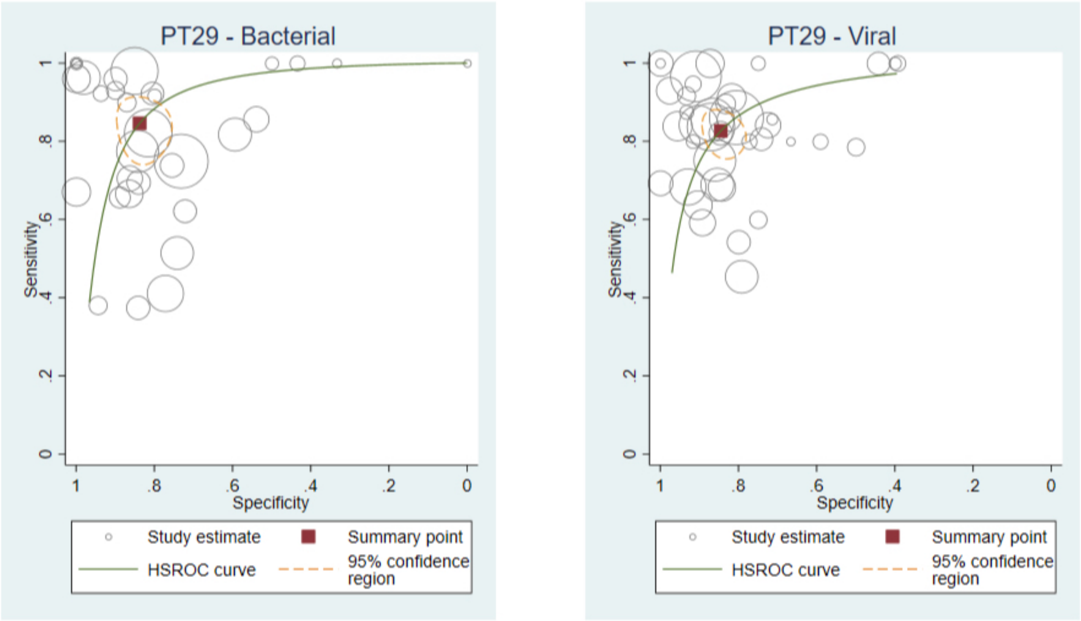

**Fig. S2. HSROC Curves for Evaluated Signatures (continued)** [11/14]

# RC31

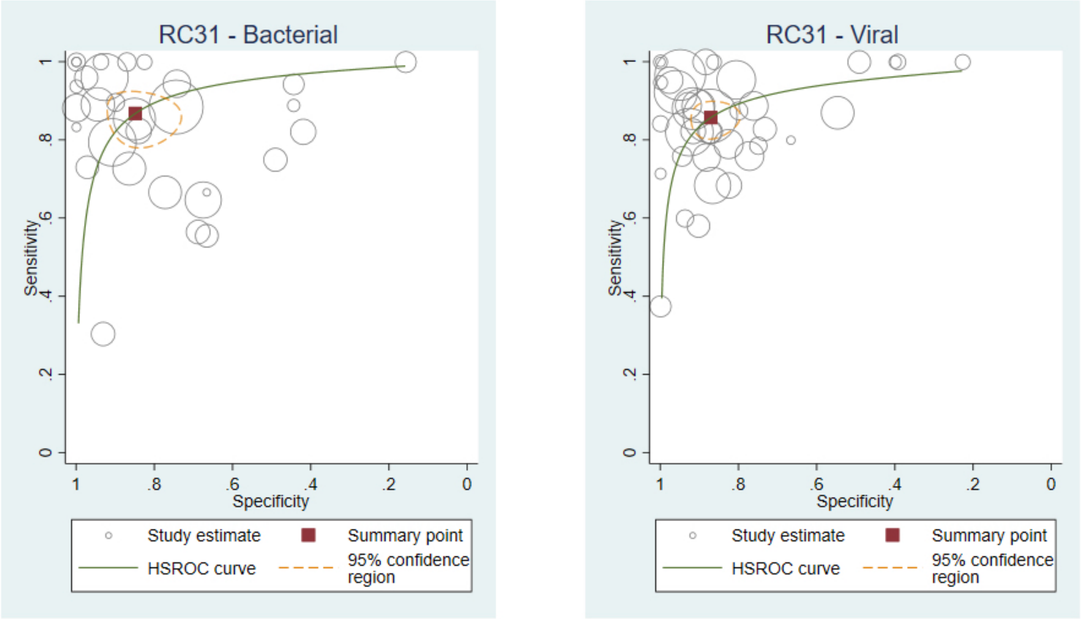


# HS33

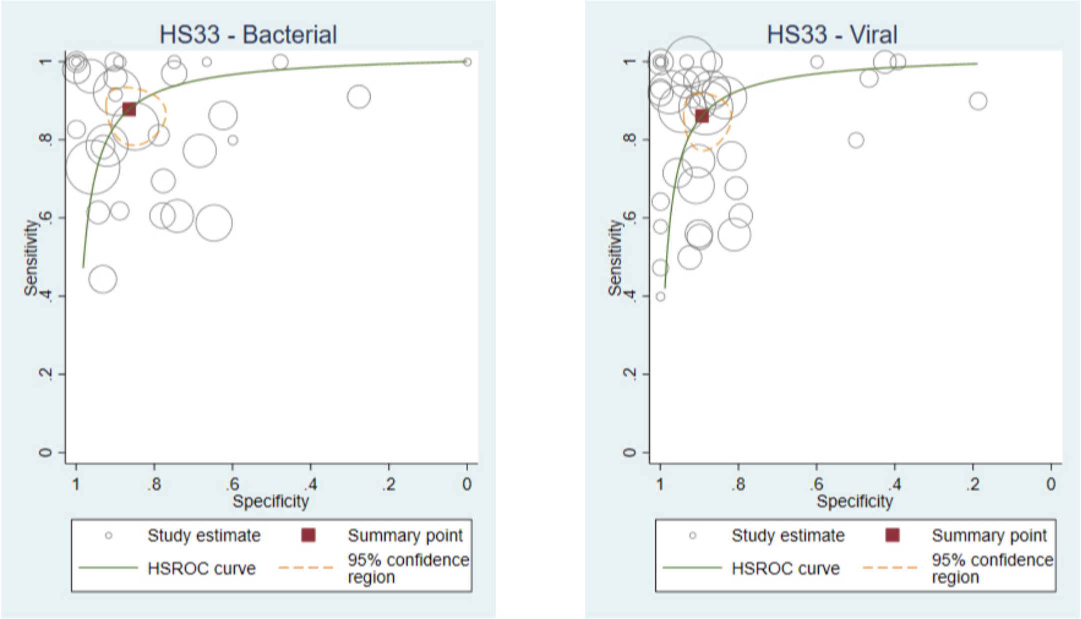

**Fig. S2. HSROC Curves for Evaluated Signatures (continued)** [12/14]

# HL34

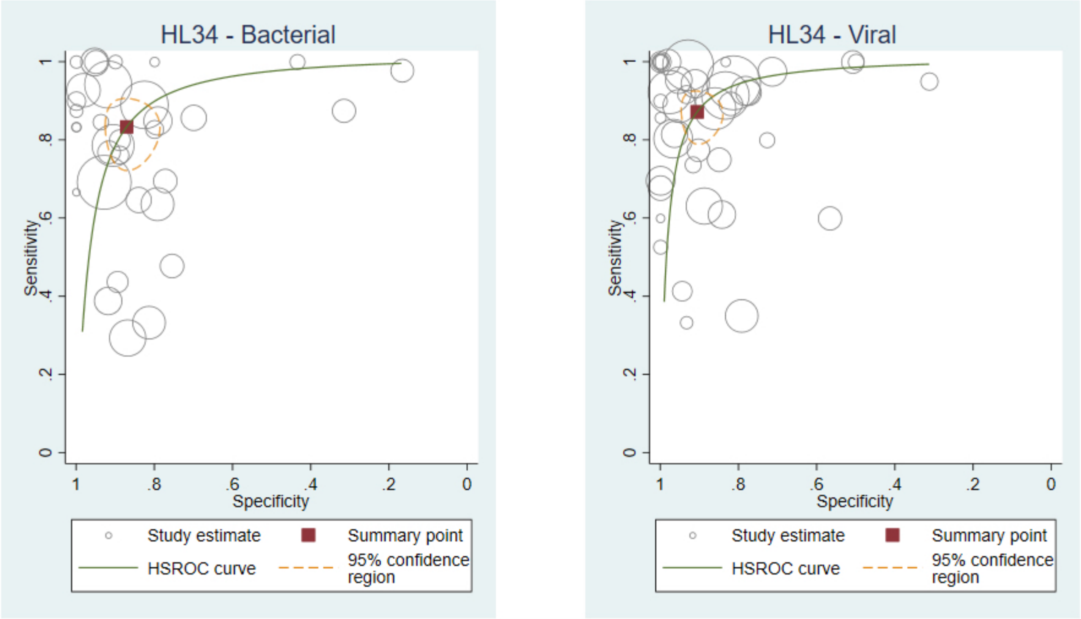


# ZG48

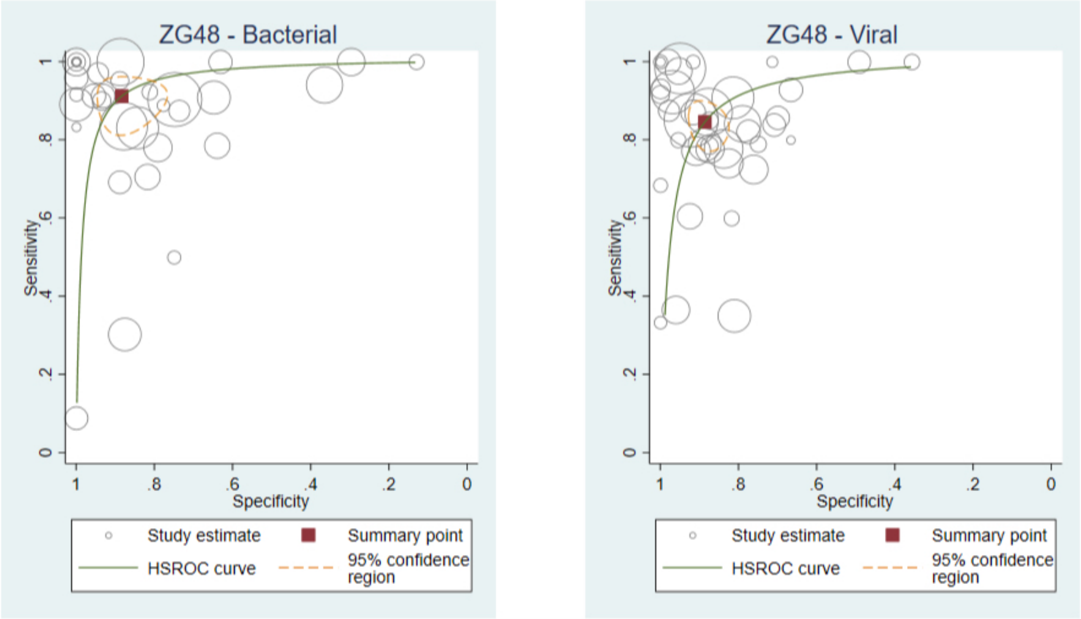

**Fig. S2. HSROC Curves for Evaluated Signatures (continued)** [13/14]

# MR59

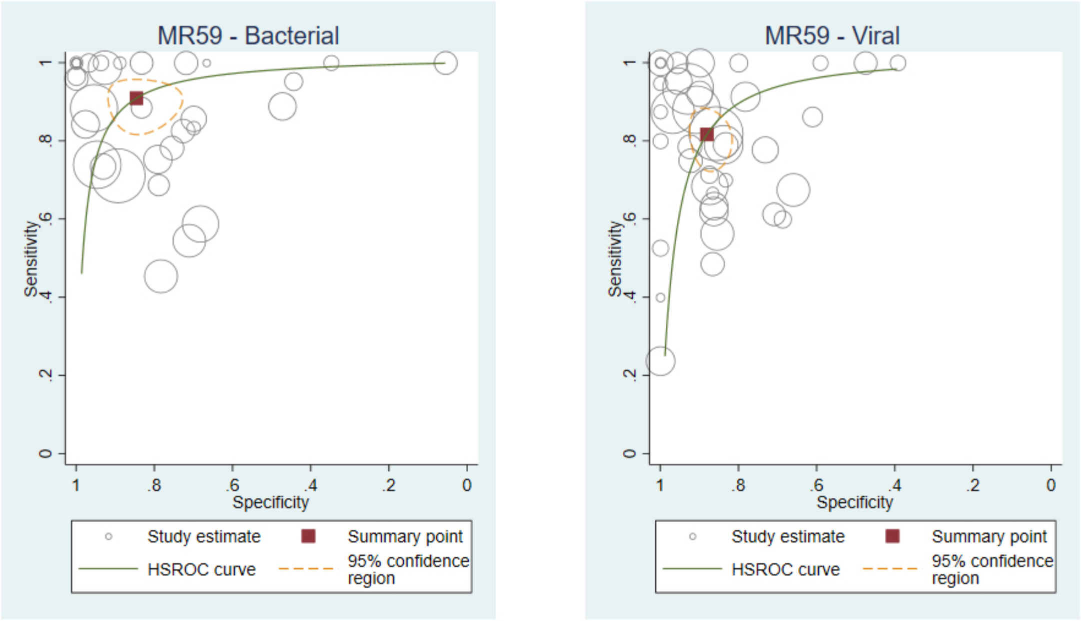


# TW96

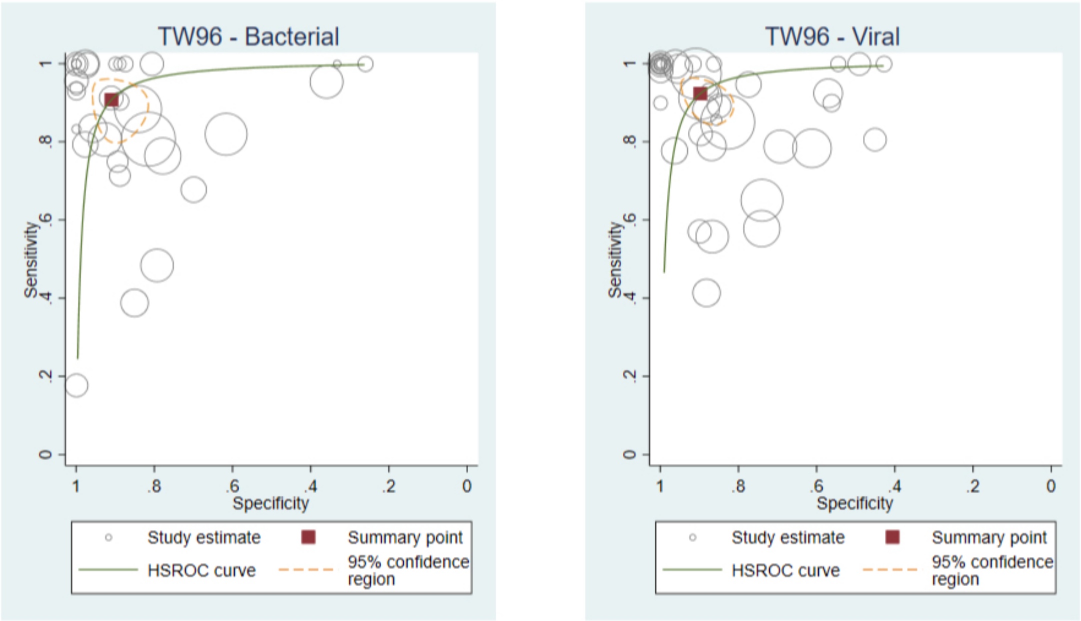

**Fig. S2. HSROC Curves for Evaluated Signatures (continued)** [14/14]

# MW139

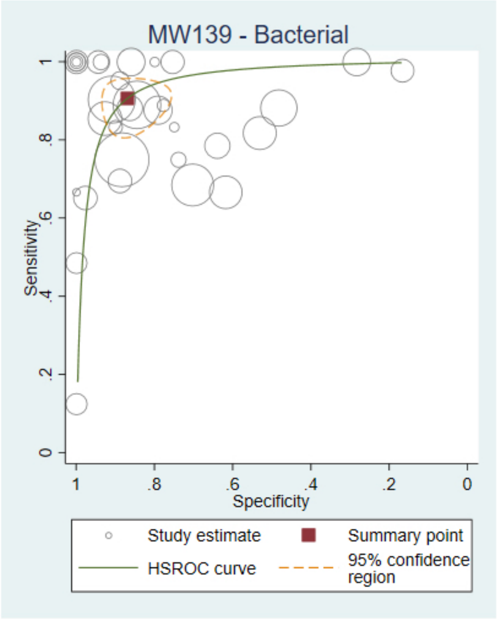


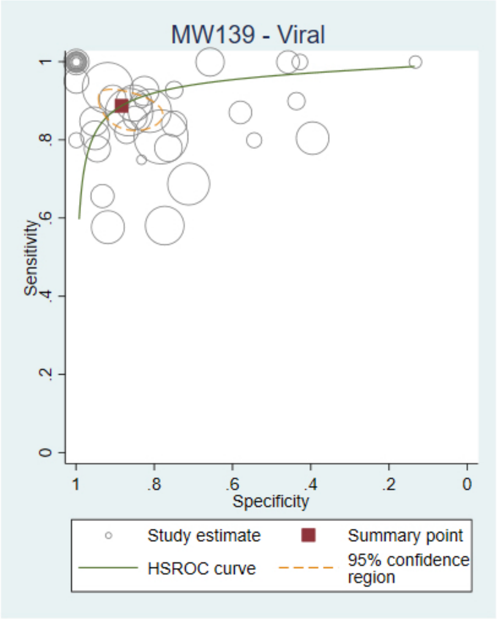


# AK398

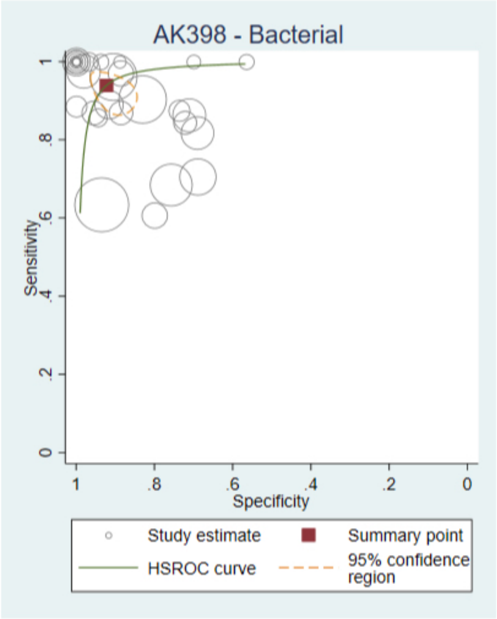


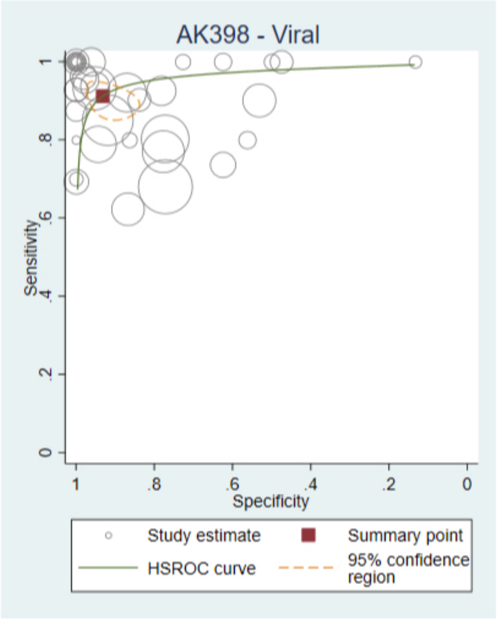

**Fig. S3. Signature Performance by Validation Dataset.** Box-plots were generated for each validation dataset's AUCs as measured across the 29 gene expression signatures for bacterial vs. non-bacterial and viral vs. non-viral classification.

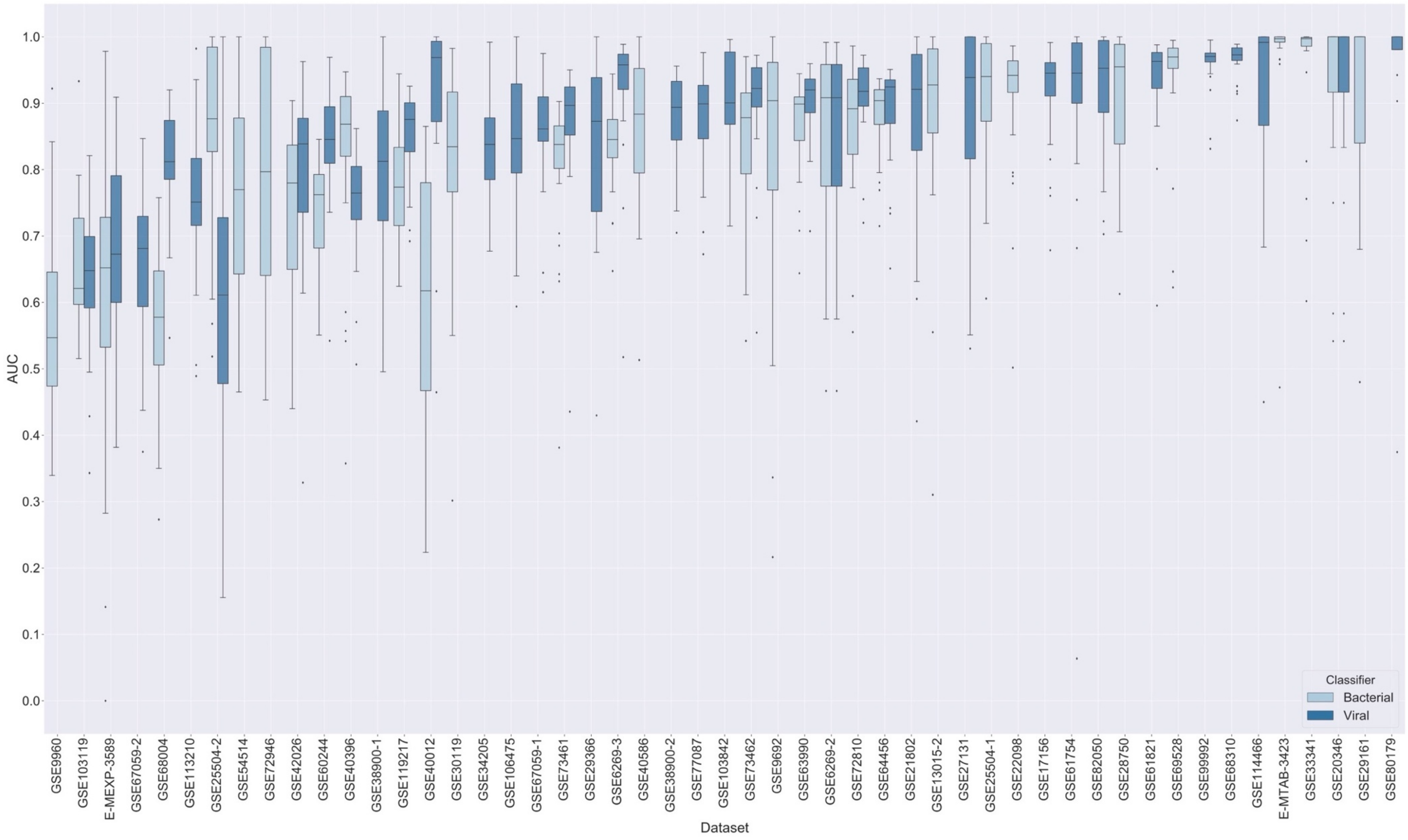

Supplement: Supplementary file 1 — Additional file 1:. Document containing supplementary tables S1-S5 and supplementary figures S1-S3 [file 13073_2022_1025_MOESM1_ESM.pdf]
